# Quantum Size-Driven Spectral Variations in Pillar[n]arene Systems: A Density Functional Theory and Wave Function Assessment

**DOI:** 10.3390/molecules29091912

**Published:** 2024-04-23

**Authors:** Cailian Yao, Tao Wang

**Affiliations:** College of Science, Liaoning Petrochemical University, Fushun 113001, China

**Keywords:** OPA, TPA, ECD, Raman, AIMD

## Abstract

This study explores the quantum size effects on the optical properties of pillar[n]arene (n = 5, 6, 7, 8) utilizing density functional theory (DFT) and wave function analysis. The mechanisms of electron transitions in one-photon absorption (OPA) and two-photon absorption (TPA) spectra are investigated, alongside the calculation of electron circular dichroism (ECD) for these systems. Transition Density Matrix (TDM) and electron–hole pair density maps are employed to study the electron excitation characteristics, unveiling a notable size dependency. Analysis of the transition electric dipole moment (TEDM) and the transition magnetic dipole moment (TMDM) reveals the electromagnetic interaction mechanism within pillar[n]arene. Raman spectra computations further elucidate vibrational modes, while interactions with external environments are studied using electrostatic potential (ESP) analysis, and electron delocalization is assessed under an external magnetic field, providing insights into the magnetically induced current phenomena within these supramolecular structures. The thermal stability of pillar[n]arene was investigated by ab initio molecular dynamics (AIMD).

## 1. Introduction

Pillar[n]arene is a novel columnar macrocyclic oligomeric supramolecular compound composed of hydroquinone or hydroquinone ether para-linked with methylene [1,2]. It has a unique rigid symmetrical structure. New macrocyclic main molecules after the classic macrocyclic molecules, such as crown ether, cyclodextrin, cucurbituril and calixarene, have gradually become an important supramolecular building block due to their highly symmetrical columnar rigid molecular structure, the variety of simple and easy functionalization methods, and their unique host–guest recognition properties [3,4,5,6]. The publication of crown ethers by Pedersen et al. in 1967 sparked interest in macrocyclic compounds [7]. It has led the development of host–guest and supramolecular chemistry for more than half a century. However, among the many large cyclic aromatic hydrocarbons, para-methylene-bridged cyclic aromatic hydrocarbons have not received much attention due to their complex synthesis. It was not until 2008 that Ogoshi et al. reported a symmetric “columnar” macrocyclic supramolecular main compound formed by para-bridging phenol-columnar aromatics [8]. Since then, this macrocyclic skeleton has been widely studied in the field of supramolecular chemistry [9,10,11,12,13].

Pillararenes are a new type of synthetic supramolecular macroring with independent cavities. Their unique host–guest chemical properties and cavity size selectively identify and capture specific small molecules. The unique structural properties and supramolecular properties of pillararenes make them an ideal base for constructing functional porous materials [14]. As a new class of symmetric calixarene analogs, pillararenes have a more rigid skeleton structure, and their symmetrical columnar structure makes it easier to construct interpenetrating polymers and tubular assemblies [15]. According to the number of units forming pillararenes, they can be divided into pillar[n]arenes. Among them, pillar[n]arenes (n = 5, 6) have been studied a great deal [16,17,18,19,20].

The synthesis of macrocyclic compounds is usually controlled by dynamics and thermodynamics. In the process of dynamics control, reaction temperature and reaction time have a great influence on the yield of macrocyclic compounds [21]. Under the control of thermodynamics, Ogoshi et al. reacted pillar[5]arene with Ethyl Bromoacetate under sodium hydride conditions, hydrolyzed by sodium hydroxide, and then formed carboxylic acid salts with aqueous ammonia, and finally obtained a water-soluble pillar[5]arene molecule. While the water solubility of pillararene molecules is very poor, this method greatly improves the water solubility of the pillar[5]arene molecules [22]. Then, they studied several reaction solvents that can adapt to the cavity of pillar[6]arene, and used them as template solvents for the synthesis of pillar[6]arene under thermodynamic control. Pillar[6]arene can hold larger volumes of hydrocarbons. They used larger hydrocarbons as template solvents for the synthesis of pillar[6]arene. Using chlorocyclohexane as a solvent, pillar[6]arene was obtained as the main product [23]. In 2012, Chen et al. reported two methods for synthesizing pillar[7]arene. One is the condensation of 1,4 dimethoxy-benzene and polyformaldehyde in CHCl_3_ catalyzed by FeCl_3_ to obtain dimethoxy-pillar[7]arene. Another method is the condensation of 2,5-bis (benzoxy methyl)-1,4-dimethoxybenzene catalyzed by p-toluenesulfonic acid in CH_2_Cl_2_ to obtain a compound demethylated with BBr3 to obtain pillar[7]arene [24].

At present, there is much research on the synthesis of pillar[n]arenes, but there are few theoretical studies on pillar[n]arenes. Based on this, we have studied the physical mechanism of the spectrum and chirality of different-sized pillar[n]arenes by first-principles calculation. Our study provides theoretical guidance for research on pillar[n]arenes in the field of optoelectronics. These molecular complexes can be used in optoelectronic devices, such as organic photodiodes, solar cells, and photoelectric sensors [25].

## 2. Results and Discussion

**Figure 1:**
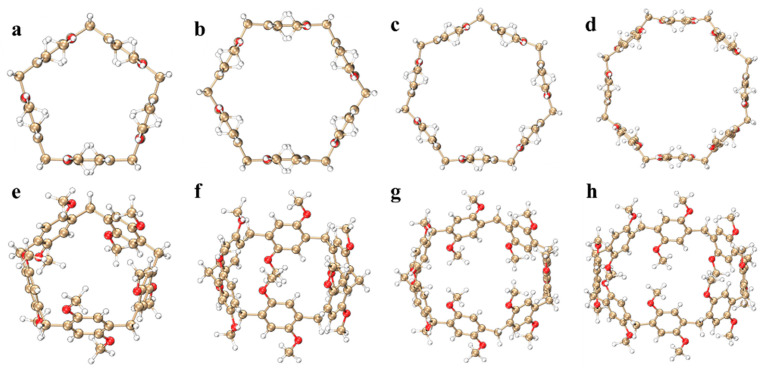
Molecular structure of pillar[n]arene, main figure: (**a**–**d**); side view: (**e**–**h**). Gold: carbon atoms, white: hydrogen atoms, red: oxygen atoms.

Pillar[n]arene is considered a key player in supramolecular chemistry due to its easy synthesis and versatility [26]; it has led to many applications in electrochemical and medical materials [27]. This article mainly draws pillar[n]arenes (n = 5, 6, 7, 8) of four different sizes, as shown in Figure 1. Pillar[n]arene consists of 1,4-dimethoxybenzene units connected by methylene bonds at positions 2 and 5 (para). The position of the methylene bridge bonds contributes to these highly symmetrical structures. It can be found that when n = 5, 1,4-dimethoxybenzene is connected to methylene and maintains a horizontal position, and when the size increases, 1,4-dimethoxybenzene gradually curves inward, and the larger the size of the molecule, the more obvious the degree of curvature. The calculated results show that the molecular pore diameters of pillar[n]arene (n = 5, 6, 7, 8) are 5.178148 Å, 6.870515 Å, 8.595161 Å, and 10.171600 Å, respectively. It can be seen that with the increase in n, the pore diameter of the molecule gradually increases, so its adsorption capacity will become stronger. The schematic diagram of the pore is shown in Supplementary Materials: Figure S1. In this section, the molecular orbitals, electronic transition properties, chiral mechanism, vibration mode, anisotropy of the induced current density, and electrostatic potential (ESP) [28] of pillar[n]arene are studied.

### 2.1. HOMO-LUMO Molecular Orbital Analysis

**Figure 2:**
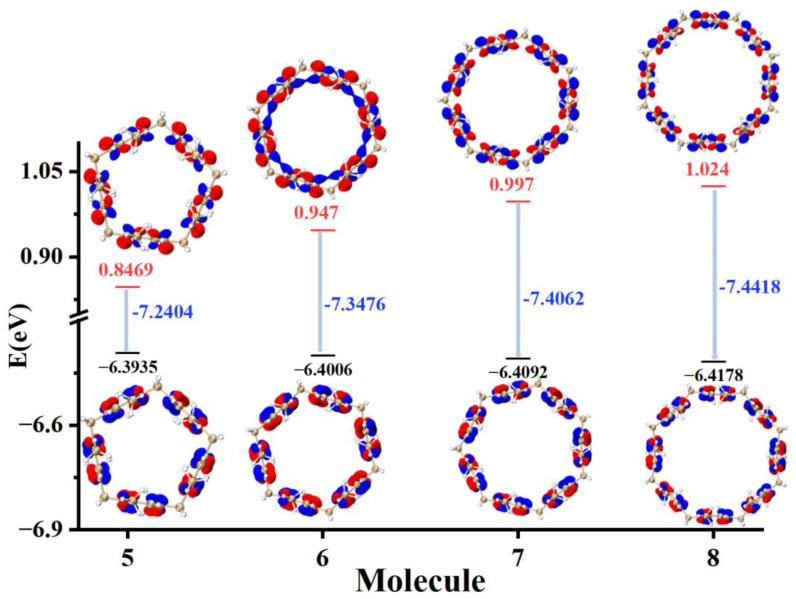
HOMO-LUMO of pillar[n]arene (n = 5, 6, 7, 8); Blue represents positive phase, red represents negative phase.

The magnitude of the highest occupied molecular orbital (HOMO) and the lowest unoccupied molecular orbital (LUMO) energies reflects the ability of the molecule to gain and lose electrons. The higher the HOMO level, the lower the ionization energy, indicating that the molecule is more likely to lose electrons. The lower the LUMO level value, the easier it is for the molecule to acquire electrons. The HOMO-LUMO gap value reflects the ability of electrons to transition from occupied orbitals to empty orbitals, and the larger the HOMO-LUMO gap value, the less likely the electron transition is to occur. The HOMO and LUMO molecular orbitals of pillar[n]arene of different sizes are drawn in Figure 2. The positive and negative phases of HOMO and LUMO are shown in blue and red, respectively. It can be seen that with the increase in n, the HOMO energy level becomes larger, the LUMO energy level becomes smaller, and the value of the HOMO-LUMO gap increases. Therefore, pillar[n]arene is less prone to electronic transition with the increase in n. By observing the molecular orbital isosurface map of HOMO-LUMO, it can be found that S_0_ → S_1_ is a local excitation, and the excitation of electrons mainly occurs in the benzene ring and O atoms, but there is no electron excitation on methylene.

### 2.2. OPA Spectrum and TPA Spectrum Analysis

**Figure 3:**
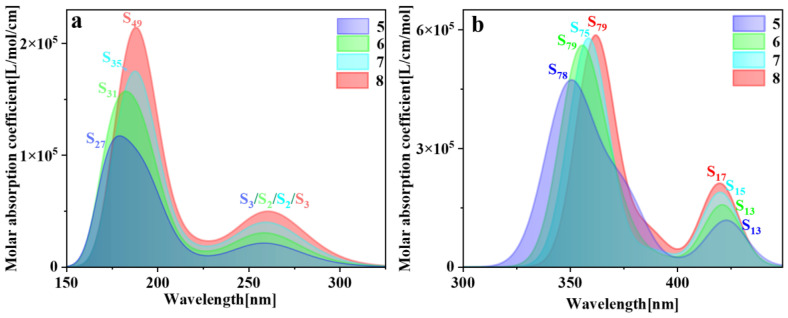
Combined figure of OPA spectrum (**a**) and TPA absorption spectrum (**b**) of pillar[n]arene.

The spectral characteristics of molecules are intricately linked to their physical structures; hence, electron transition characteristics can be deduced through spectral analysis. The higher the oscillator strength between the ground state and the excited state structures, the greater the propensity for absorbing electromagnetic waves of matching frequencies and transitioning to the excited state. Consequently, the absorption peaks in the spectrum also exhibit greater intensity. Typically, comparison with electronic energy spectra and experimental data is imperative [29]. So, we plotted the one-photon absorption (OPA) and two-photon absorption (TPA) spectra of pillar[n]arenes of different sizes (refer to Figures S2 and S3). Figure 3 is the combined figure of the two absorption spectra. The OPA spectrum in Figure 3a shows that the absorption spectrum band of pillar[n]arene is mainly between 150 and 350 nm. Pillar[n]arenes of different sizes all have two absorption peaks. With the increase in n, the absorption spectrum will redshift and the absorption intensity will increase with the increase in molecular size. So, the size dependence of the absorption spectrum is very regular. This is because the width between the electron-occupied molecular orbital energy level and the unoccupied molecular orbital energy level decreases due to the increase in the size of the molecule, and the energy gap becomes smaller, so the absorption moves in the direction of the long wave. Figure 3b is the TPA spectrum, which has twice the absorption wavelength of the OPA spectrum, so the absorption range is between 325 and 450 nm. Pillar[n]arenes of different sizes have two absorption peaks. The strongest absorption peak redshifts as n increases. The weaker absorption peak on the right side blueshifts with the increase in n, and the absorption intensity increases with the increase in size.

#### 2.2.1. One-Photon Transition Characteristics

**Figure 4:**
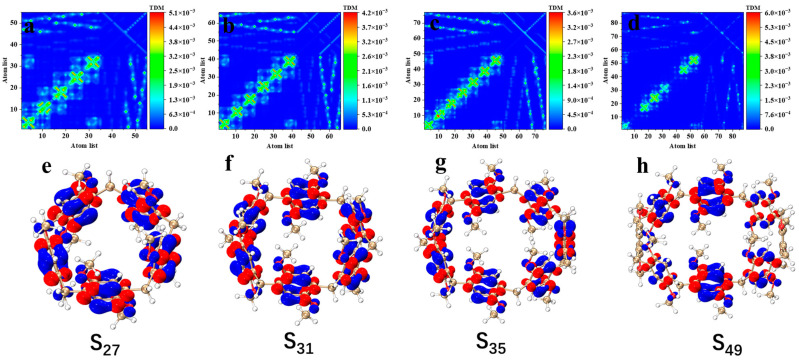
Pillar[n]arene’s TDM and electron–hole pair density (**e**–**h**) of the excited states S_27_ (**a**), S_31_ (**b**), S_35_ (**c**), and S_49_ (**d**) at the main absorption peak near 150–200 nm, respectively.

The study of the electron transition mechanism helps to reveal the behavior of molecules in spectroscopy and provides a basis for the design and optimization of optoelectronic devices [30]. To further analyze the excited-state characteristics of the electron spectrum, the transition density matrix (TDM) (Figure 4a–d) and electron–hole pair densities (Figure 4e,f) of the excited states that contribute the most to the main absorption peak in pillar[n]arenes of different sizes were plotted (the red isosurface represents the area where electrons increase, and the blue isosurface represents the area where electrons decrease). As shown in Figure 4, when the absorption peak is around 150–200 nm, the TDM diagram of excited states corresponding to the pillar[n]arene peak has very similar characteristics. The transition density is mainly concentrated on the diagonal line, with a small distribution in the upper left and lower right corners, which indicates that the excitation process of pillar[n]arene is local excitation accompanied by a small amount of charge transfer. By drawing the electron–hole pair density map, it can be seen that the electrons and holes in pillar[n]arene are mainly distributed on the benzene ring, but not on methylene, indicating that local excitation occurs on the benzene ring, and the methylene bonds connecting the benzene ring do not participate in electron excitation. And the O atom has a charge transfer that transfers electron to the benzene ring. Pillar[8]arene’s electrons and holes only appear on the upper and lower dimethoxybenzenes, and there is no distribution of electrons and holes on the left and right dimethoxybenzenes, as shown in Figure 4h. These results indicate that the electron excitation of pillar[8]arene is mainly contributed by the upper and lower six dimethoxybenzenes. Figure S4 shows the TDM and electron–hole pair density of excited states at the absorption peak of pillar[n]arene near 230–300 nm. The characteristics of the TDM plot are similar to those of the TDM plot corresponding to the excited state of the main absorption peak. The transition density is mainly concentrated on the diagonal line, with a small distribution in the upper left and lower right corners, which shows that the excitation process is local excitation accompanied by a small amount of charge transfer, but the distribution of the transition density becomes sparse. By mapping the electron–hole pair density, the distribution of electrons and holes in pillar[n]arene is significantly lower than that at the main absorption peak when the absorption peak is near 230–300 nm. It shows that the excitation intensity caused by the absorption peak is weak.

In order to quantitatively analyze the excitation characteristics of electrons, we performed a wave function analysis of the excited states. Table 1 and Table S1 show the main excited state transition index of pillar[n]arene on the two absorption peaks, respectively. Vibrator strength is a dimensionless quantity, $f=\frac{2}{3}\;\Delta E{\left|\langle i\langle -r\rangle j\rangle \right|}^{2}$, and $\left|\langle i\langle -r\rangle j\rangle \right|$ is the electric dipole moment of the transition between the two states. It can be seen that the oscillator strength of the system increases significantly with the increase in n, indicating that the absorption intensity of the system can be enhanced with the increase in the size.

The H index represents the overall average width of the distribution of electrons and holes:

(1) $$H\;index=(\left|\sigma_{ele}\right|+\left|\sigma_{hole}\right|)/2$$

$\left|\sigma_{ele}\right|$ and $\left|\sigma_{hole}\right|$ are the spatial distributions of electrons and holes, respectively. The H index of pillar[n]arene increases with the increase in n; that is, with the increase in molecular size, the spatial distribution of electrons and holes becomes wider, which is consistent with the result of electron–hole pair density. Then, the excitation properties of the electron are measured by calculating the distance between the centroid of the electron and the hole and the degree of coincidence of the electron–hole, namely, the D index and the $S_{r}$ index.

(2) $$D_{X}=\left|X_{\mathrm{ele}}-X_{hole}\right|\;D_{y}=\left|Y_{\mathrm{ele}}-Y_{hole}\right|\;D_{Z}=\left|Z_{\mathrm{ele}}-Z_{hole}\right|$$

(3) $$Dindex=\sqrt{{\left(D_{X}\right)}^{2}+{\left(D_{y}\right)}^{2}+{\left(D_{z}\right)}^{2}}$$

(4) $$S_{r}index=\int S_{r}\left(r\right)dr\equiv \int \sqrt{\rho^{hole}(r)}dr$$

As you can see, pillar[n]arene has a small D index and a large $S_{r}$ index. The results show that the hole and electron distributions are not clearly separated, and the distribution width is similar, which is local excitation. Contrary to the degree of electron–hole coincidence, the degree of electron–hole separation, that is, the T index, is the difference between the D index and the average degree of extension of electrons and holes in the *CT* direction, as shown in Formula (5).

(5) $$t\;index=D\;index-H_{CT}$$

The T index of all systems is clearly negative, indicating that the degree of electron–hole separation is small, which corresponds to the large $S_{r}$ index, reflecting the local excitation property of the excited state.

#### 2.2.2. Two-Photon Transition Characteristics

Two-photon absorption is a typical third-order nonlinear optical effect, which is a process in which a substance absorbs two photons at the same time and transitions from the ground state to the excited state through an intermediate virtual state under the excitation of strong light. TPA spectra of molecules often have a wide absorption range and a strong absorption efficiency. The calculation of two-photon absorption spectra can provide information about the absorption characteristics of materials under two-photon excitation, including TPA cross section, peak, and peak position, which is of great significance for understanding the optical properties of a material.

**Figure 5:**
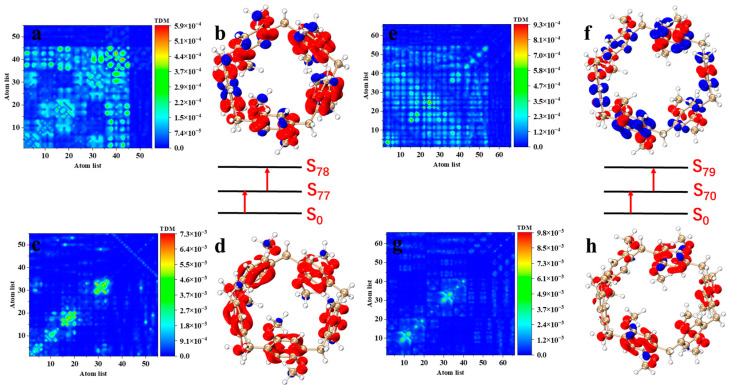
Pillar[5]arene two-step transition process in S_78_, from ground state to intermediate state (**c**) and from intermediate state to final state (**a**), and their TDM and electron–hole pair density (**b**,**d**); pillar[6]arene two-step transition process in S_79_, from the ground state to the intermediate state (**g**) and from the intermediate state to the final state (**e**), and their TDM and electron–hole pair density (**f**,**h**). The red isosurface represents the electron; The blue isosurface represents the hole.

As with one-photon absorption, we study the excitation properties of TPA by TDM and electron–hole pair density. In the TPA spectrum of Figure 3b, pillar[5]arene has the strongest two-photon cross section in the excited state of S_78_, and the intermediate state of S_78_ is S_77_. The TMD diagram of S_0_–S_77_ (Figure 5c) shows that the transition density is mainly distributed diagonally, but also in the upper left and lower right corners. In the electron–hole pair density diagram (Figure 5d), electrons are distributed on dimethoxy-benzene, while holes are only distributed on the C atom of the methoxy. The separation of electrons and holes is significant, which represents the excitation property of charge transfer. The TMD diagram of S_77_–S_78_ (Figure 5a) shows that the transition density is mainly distributed in the lower left part. In the electron–hole pair density diagram (Figure 5b), electrons are concentrated on the C atom and O atom of the benzene ring, while the holes are distributed on the C atom without connecting functional groups and the C atom of methoxy in the benzene ring, which also belongs to charge transfer excitation. The degree of electron transfer in the second step is stronger than that in the first step. The strongest two-photon cross section of pillar[6]arene is contributed by S_79_. The intermediate state of S_79_ is S_70_. The first transition of pillar[6]arene is local excitation accompanied by charge transfer excitation. The second transition belongs to charge transfer excitation. The electrons are concentrated on the C and O atoms connected to the methoxy, and the holes are concentrated on the other C atoms of the benzene ring; refer to Figure 5e–h.

**Figure 6:**
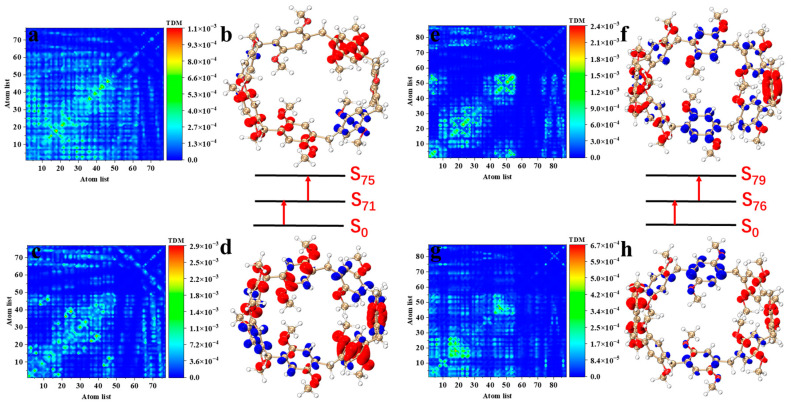
Pillar[7]arene two-step transition process in S_75_, from ground state to intermediate state (**c**) and from intermediate state to final state (**a**), and their TDM and electron–hole pair density (**b**,**d**); pillar[8]arene two-step transition process in S_79_, from the ground state to the intermediate state (**g**) and from the intermediate state to the final state (**e**), and their TDM and electron–hole pair density (**f**,**h**). The red isosurface represents the electron; The blue isosurface represents the hole.

Figure 6a–d show the TMD diagram and electron–hole pair density diagram of pillar[7]arene in the S_75_ excited state. The first step of the transition S_0_–S_71_ belongs to charge transfer excitation, and the electrons on the C atom in the benzene ring that are not connected to the methoxy are transferred to the C atom and the O atom that are connected to the methoxy in the excitation process; refer to Figure 6c,d. During the second step transition, electrons are distributed on the benzene ring in the lower left and upper right parts, and holes are distributed in the lower right part. The electron–hole separation degree is high, and the charge transfer characteristics are significant, as shown in Figure 6a,b. The maximum TPA cross section of pillar[8]arene is contributed by S_79_. The first and second transitions also belong to charge transfer excitation. Electrons are transferred from the upper and lower benzene rings to the left and right sides of the molecule during excitation; refer to Figure 6e–h.

Table 2 shows the transition dipole moments of the major TPA-excited states of pillar[n]arene. The displacement of the TPA spectrum is related to the transition dipole moments of molecules. With the increase in size, the dipole moment of the molecule increases significantly, resulting in a large absorption cross section. Moreover, the transition dipole moment from the ground state to the intermediate state decreases with the increase in the size, and the transition dipole moment from the intermediate state to the final state increases with the increase in the size. The charge transfer in the second step transition is significantly enhanced, so the total energy required for molecular excitation decreases, resulting in the absorption spectrum redshift.

We also drew the TMD diagram and the electron–hole pair density diagram of the excited two-photon state corresponding to the absorption peak of pillar[n]arene at 400–450 nm, as shown in Figures S5 and S6. Figure S5a–d show the TMD diagram and electron–hole pair density diagram of pillar[5]arene in the S_13_ excited state. The first step of the transition S_0_–S_2_ belongs to local excitation; during the second step of the transition, electrons are distributed on the C atom that is not connected to the methoxy in the benzene ring, and the holes are distributed on the C atom and the O atom that is connected to the methoxy. The electron–hole separation degree is high, and the charge transfer characteristic is remarkable. The two-photon absorption cross section of pillar[6]arene at 400–450 nm is contributed by S_13_. The first step transition belongs to local excitation, and the second step transition belongs to charge transfer excitation. The transfer characteristics are similar to pillar[5]arene (see Figure S5e–h). Figure S6 shows the TMD diagram and electron–hole pair density diagram of the excited two photons corresponding to the absorption peaks of pillar[7]arene and pillar[8]arene at 400–450 nm, which are the same as the absorption characteristics of pillar[5]arene. When the absorption peak is between 400 and 450 nm, the electron transfer degree of the first step transition is stronger than that of the second step.

Table S2 shows the transition dipole moment of the excited two-photon absorption state of pillar[n]arene between 400 and 450 nm. The dipole moment of the molecule increases obviously with the increase in the size of pillar[n]arene, resulting in a large absorption cross section. Different from the main absorption peak, the transition dipole moment from the ground state to the intermediate state increases with the increase in the size, and the transition dipole moment from the intermediate state to the final state decreases with the increase in the size. The charge transfer of the second step transition weakens, and the total energy required for molecular excitation increases, resulting in the blueshift of the absorption spectrum.

### 2.3. Chiral Physical Mechanism of ECD

**Figure 7:**
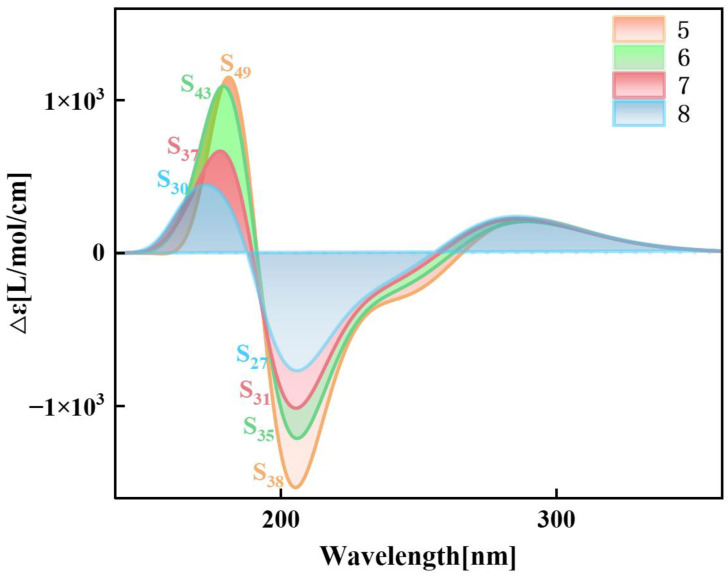
ECD spectrum of pillar[n]arene.

**Figure 8:**
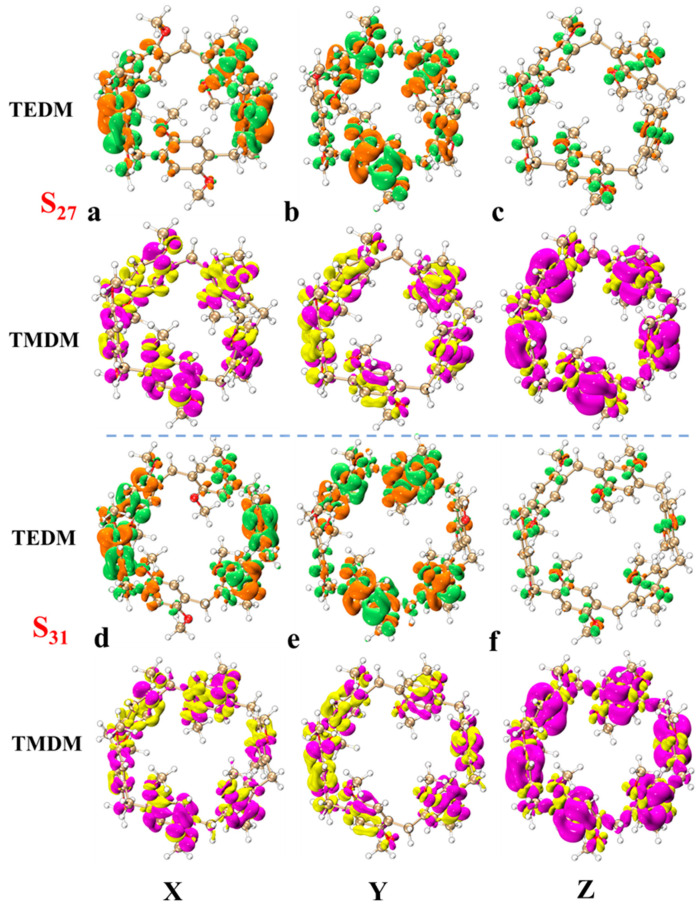
TEDM and TMDM of major ECD excited states of pillar[5]arene and pillar[6]arene near 190–230 nm. (**a**) pillar[5]arene’s TEDM and TMDM in the X direction; (**b**) pillar[5]arene TEDM and TMDM in the Y direction. (**c**) pillar[5]arene in the Z direction of TEDM and TMDM. (**d**–**f**) Column [6] aromatics in the X, Y, Z direction of TEDM and TMDM respectively (Positive and negative isosurfaces of TEDM are represented by green and orange isosurfaces; the positive and negative isosurfaces of TMDM are represented by purple and yellow isosurfaces).

**Figure 9:**
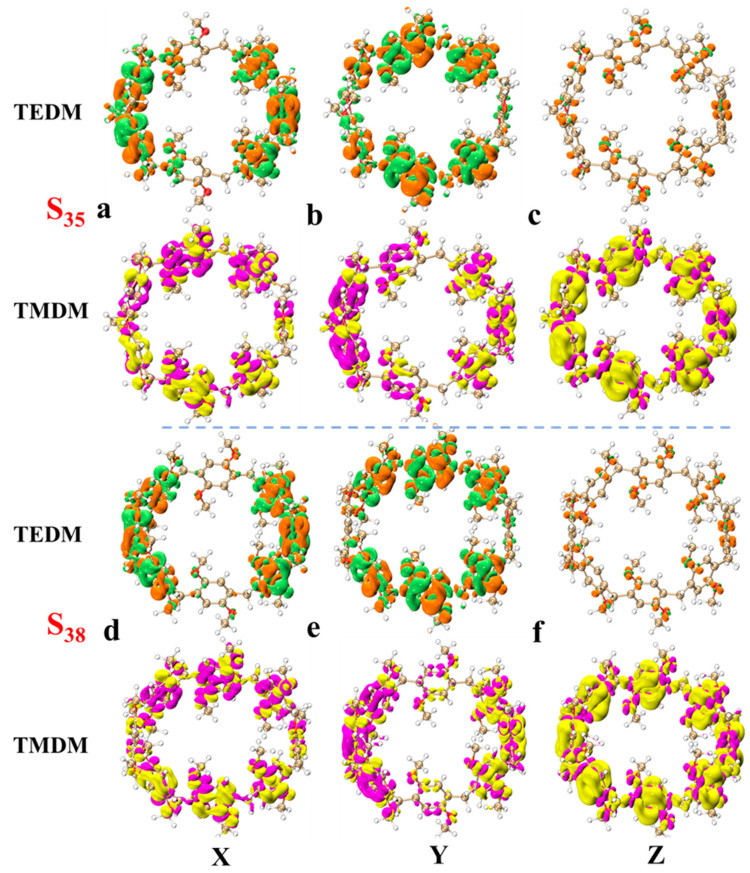
TEDM and TMDM of major ECD excited states of pillar[7]arene and pillar[8]arene near 190–230 nm. (**a**) pillar[7]arene’s TEDM and TMDM in the X direction; (**b**) pillar[7]arene TEDM and TMDM in the Y direction. (**c**) pillar[7]arene in the Z direction of TEDM and TMDM. (**d**–**f**) Column [8] aromatics in the X, Y, Z direction of TEDM and TMDM respectively (positive and negative isosurfaces of TEDM are represented by green and orange isosurfaces; The positive and negative isosurfaces of TMDM are represented by purple and yellow isosurfaces).

**Figure 10:**
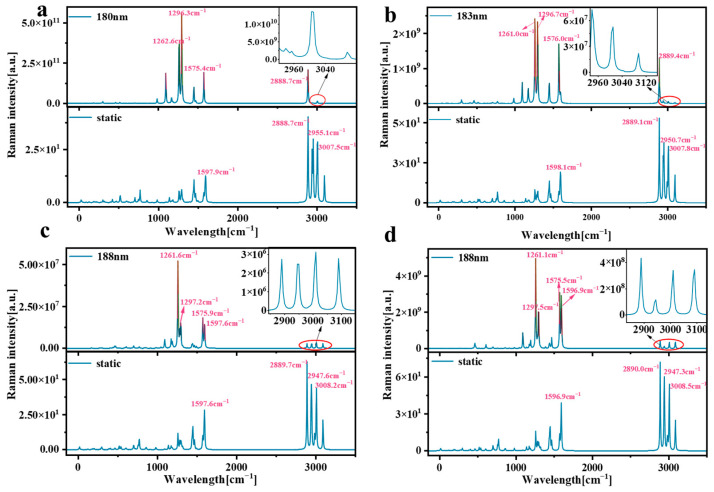
Raman and resonance Raman spectra of pillar[n]arene. (**a**) pillar[5]arene; (**b**) pillar[6]arene; (**c**) pillar[7]arene; (**d**) pillar[8]arene.

**Figure 11:**
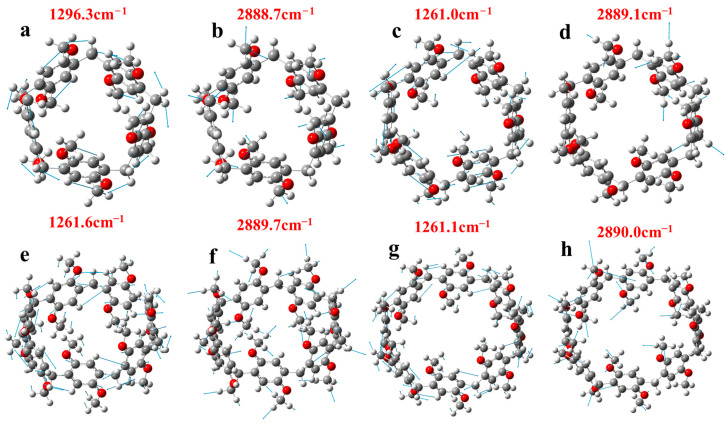
Vibration modes of pillar[n]arene at the peak of Raman absorption.

In order to understand the light absorption characteristics of pillar[n]arene more clearly, we calculated the chiral characteristics. Chirality is one of the conformational differences in the system. When left- and right-handed circularly polarized light passes through chiral molecules, its polarization direction rotates. This phenomenon is called circular dichroism. Circular dichroism provides information about the three-dimensional structure and chiral properties of molecules, which are crucial for drug design, chiral catalysis, and biomolecular research. By measuring electron circular dichroism (ECD), information about molecular structure can be obtained [31,32]. Therefore, we calculated the ECD spectrum of pillar[n]arene, as shown in Figure S7, and a combined figure of the ECD spectrum is shown in Figure 7. The four structures have two positive absorption peaks and one negative absorption peak between 150 and 350 nm. With the increase in molecular size, the absorption peak is redshifted, and the rotatory strength decreases with the increase in n. Therefore, the larger the size of pillar[n]arene, the stronger the chirality. Next, by calculating the transition electric dipole moment density (TEDM) and transition magnetic dipole moment density (TMDM) of the excited state corresponding to the strong absorption peak, we further analyze the difference in the circular dichroism of pillar[n]arenes of different sizes. Figure 8 shows the TEDM/TMDM of excited states corresponding to negative absorption peaks of pillar[5]arene in different directions. S_27_ makes the largest contribution to its peak value, and the circular dichroism is negative. TEDM on the X component is mainly distributed on the benzene ring on both sides of the molecule, and the positive and negative equivalences are symmetrical, as shown in Figure 8a. The TEDM in the Y component is mainly distributed in the upper and lower parts of the molecule, as shown in Figure 8b. The distribution TEDMs on the Z component is very small, and almost all of them are positive isosurfaces; refer to Figure 8c. The distribution of TMDM is complementary to TEDM to a certain extent, and the position where there is no TEDM isosurface is the position of the TMDM isosurface, because the direction of electricity and magnetism in electromagnetic wave is vertical; refer to Figure 8a–c. Pillar[5]arene’s TMDM in the Z direction is mainly a positive isosurface (see Figure 8c). The molecular distribution characteristics of TEDM and TMDM of pillar[6]arene in S_31_ are similar to those of pillar[5]arene; see Figure 9a–c. In Figure 10c and Figure 11c, pillar[7]arene and pillar[8]arene have a small number of negative isosurfaces distributed on TEDM in the Z direction. TMDM and TEDM are complementary, and the negative isosurfaces are distributed on almost the entire molecule.

Figures S8 and S9 show the TEDM/TMDM in different directions of the excited state corresponding to the positive absorption peaks of pillar[n]arene near 150–190 nm. The TEDM of pillar[5]arene in the X direction of S_30_ still has left and right distribution, the TEDM in the Y direction has up and down distribution, and there is less distribution in the Z direction, and the positive and negative values are separated. TMDM and TEDM are complementary. The positive isosurface of TMDM in the Z direction is concentrated in the right half, and the negative isosurface is concentrated in the left half, as shown in Figure S8a–c. The TEDM and TMDM distributions of pillar[6]arene in S_37_ are very similar to those of pillar[5]arene; see Figure S8d–f. Pillar[7]arene in the Z direction of S_43_ has a positive isosurface on the left and a negative isosurface on the right. See Figure S9a–c for reference. Pillar[8]arene in the Z direction of S_49_ has a positive isosurface on the top and a negative isosurface on the bottom half. See Figure S9d–f. The positive and negative region separation of TMDM in the Z direction indicates that molecular polarization has a great influence on the TMDM of the system.

The absolute value of the tensor product of the molecule represents the intensity of optical excitation, and the greater the absolute value of the tensor product, the stronger the intensity of excitation. Table 3 shows the TEDM/TMDM values of each excited state corresponding to the negative peak value of pillar[n]arenes of different sizes at 190–230 nm and the eigenvalues of the sum tensor product. It can be seen from the table that with the increase in molecular size, the absolute value of the tensor product increases significantly, and the values of the product tensor are all negative, which fully matches the strength and direction of ECD. Table S3 shows the TEDM/TMDM values of each excited state corresponding to the positive absorption peak near 150–190 nm for pillar[n]arenes of different sizes and the eigenvalues of the sum tensor product. The values of the product tensor are all positive and increase with the increase in molecular size, corresponding to the ECD spectrum.

### 2.4. Raman Spectrum Analysis

Raman spectroscopy is a very useful tool that provides information about molecular structure, vibration, and symmetry. By studying the Raman spectra of molecules, we can understand the vibration patterns inside molecules and the interactions between molecules. This is of great significance for understanding the properties of molecular materials [33]. Resonance Raman spectroscopy enhances the strength of Raman signals through the resonance effect, improves the sensitivity and detection ability of the signals, and has wide application potential in chemistry, biomedicine, material science, and other fields. We plotted the corresponding wavelength of pillar[n]arene at the position of the strong absorption peak as the excitation source for Raman spectral analysis. Figure 10 shows the Raman and resonance Raman spectra of pillar[n]arene. The strong Raman peak in the static Raman spectrum is concentrated in the position of 2880–3010 cm^−1^, and there is also a Raman peak near 1200–1600 cm^−1^. The peak value of Raman peak increases with the increase in molecular size. In the resonance Raman spectrum, the stronger Raman peaks are mainly between 1200 cm^−1^ and 1600 cm^−1^, and the smaller peaks are between 2880 and 3010 cm^−1^. It can be seen from the figure that the resonance Raman spectrum after laser irradiation has the same Raman peak wave number as the static Raman spectrum, but the Raman intensity is significantly enhanced, especially at the absorption peak Raman intensity from 1200 cm^−1^ to 1600 cm^−1^. The results show that the use of a laser can significantly enhance the Raman strength of pillar[n]arene. The 180 nm laser can increase the Raman strength of pillar[5]arene by about 10 orders of magnitude; the 183 nm laser can increase the Raman strength of pillar[6]arene by about 8 orders of magnitude, and the 188 nm laser can increase the Raman strength of pillar[7]arene and pillar[8]arene by 6 and 7 orders of magnitude, respectively.

We plotted the vibration modes at positions with strong absorption peaks in the static Raman spectra and resonance Raman spectra of pillar[n]arene, respectively, as shown in Figure 11. The resonance Raman spectrum at 1296.3 cm^−1^ has the highest peak value, and its vibration mode is the contraction vibration of the C atom and O atom and the left and right vibration of the H atom, as shown in Figure 11a. The peak value of the static Raman spectrum at 2888.7 cm^−1^ is the highest, and its vibration state is the stretching vibration of the H atom on methoxy, as shown in Figure 11b. The vibration modes of other absorption peaks are shown in Figure S10. Figure 11c,d are the vibration modes of pillar[6]arene. The resonance Raman spectrum peak value at 1261.0 cm^−1^ is the highest, and its vibration mode is the contraction vibration of atom C and atom O and the up and down vibration of atom H (refer to Figure 11c). The peak value of the static Raman spectrum at 2889.1 cm^−1^ is the highest, and its vibration state is the stretching vibration of H atom on methoxy; refer to Figure 11d. Figure 11e,f are pillar[7]arene’s vibration modes. The peak value of the resonance Raman spectrum at 1261.6 cm^−1^ is the highest, and the peak value of the static Raman spectrum at 2889.7 cm^−1^ is the highest, and its vibration state is the same as pillar[6]arene’s vibration mode. Figure 11g,h are the vibration modes of pillar[8]arene. The peak value of the resonance Raman spectrum at 1261.1 cm^−1^ is the highest, and the peak value of the static Raman spectrum at 2890.0 cm^−1^ is the highest, and the vibration mode is the same as pillar[6]arene. The vibration modes of the remaining Raman spectral absorption peaks are referred to in Figures S11–S13.

### 2.5. ESP Analysis

**Figure 12:**
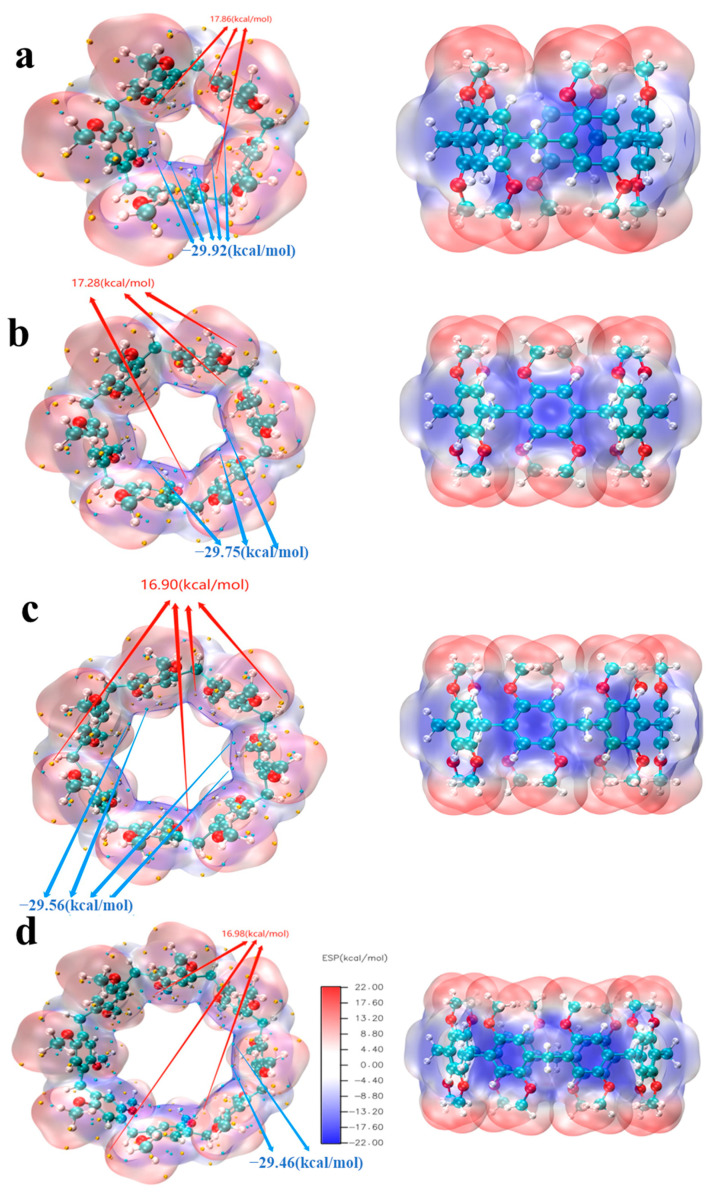
ESP distribution of pillar[n]arene. (**a**) pillar[5]arene; (**b**) pillar[6]arene; (**c**) pillar[7]arene; (**d**) pillar[8]arene.

Through ESP, we can easily and intuitively understand where the molecule tends to interact with substances (or locally) with what charge, and it is often used in the study of interactions between biomolecules, drug molecules, and other macromolecules. The ESP of the four structures is shown in Figure 12. The red isosurface represents the positive ESP, and the blue isosurface represents the negative ESP. It can be seen from the figure that the ESP near the benzene ring and the O atom is negative, while the ESP near the C and H atoms on the methoxy group is positive. The ESP minimum of pillar[n]arene is distributed in the inner part of the C ring, and the minimum decreases with the increase in the size. The maximum also decreases with increasing size (except pillar[8]arene). Therefore, the larger the molecular size, the less likely the molecular interaction is to occur.

### 2.6. Response of Pillar[n]arene to External Magnetic Field and Magnetically Induced Current Density

**Figure 13:**
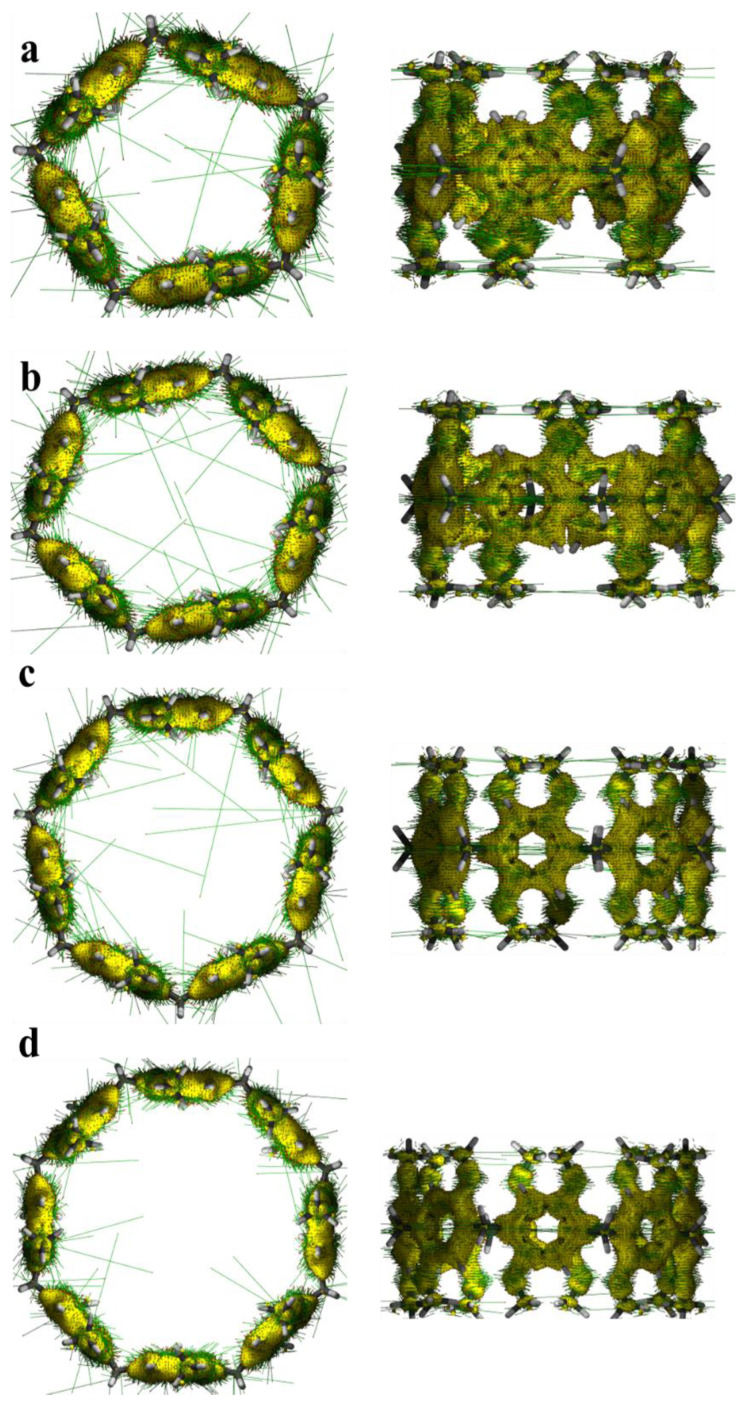
Main and side views of the external magnetic field direction and magnetic-induced current density of pillar[n]arene. The direction of the external magnetic field is perpendicular to the ring plane, the yellow isosurface is the current density, and the green line on the isosurface is the current direction. (**a**) pillar[5]arene; (**b**) pillar[6]arene; (**c**) pillar[7]arene; (**d**) pillar[8]arene.

For a molecular system, if the electrons in the system have a strong delocalization in the whole of each electron or in a certain part, then when the magnetic field is applied, an obvious induced ring current will be generated in the corresponding region, and there will be a larger current density near the relevant atoms. After applying an external magnetic field to the pillar[n]arene system, we found that each benzene ring and methoxy formed a rich magnetic-induced current (Figure 13), while there was no magnetic-induced current density around the H atoms and methylene. This is because the H atom has only one electron, which will be used to bond with carbon, and the C atom on methylene has no delocalized π electron. There are abundant delocalized π electrons in the benzene ring, which are highly responsive to the external magnetic field.

### 2.7. Molecular Dynamics Analysis

**Figure 14:**
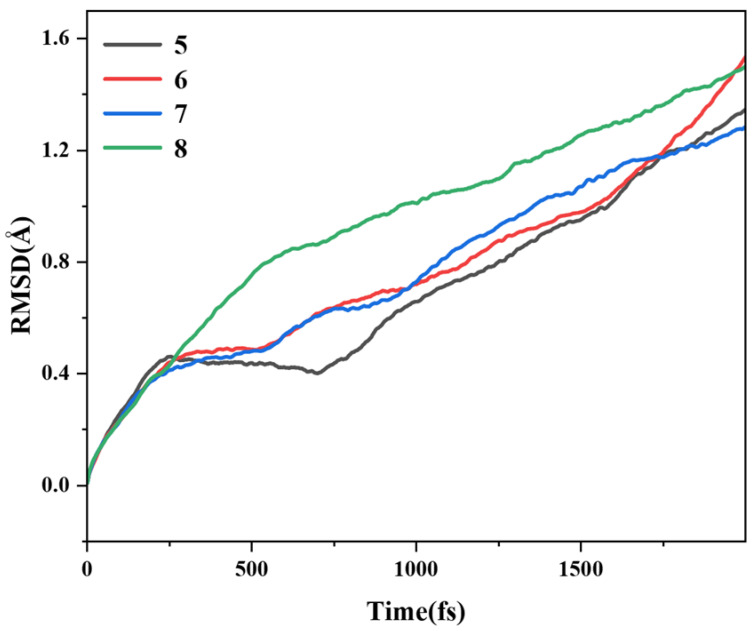
RMSD of pillar[n]arene at 298.15 K.

Based on AIMD, the thermal stability of four pillar[n]arene compounds was investigated at 298.15 K (room temperature) to compare the stability regimes of several structures. The motion trajectories of pillar[n]arene were first simulated in vacuum at a temperature of 298.15 K for 2000 fs, and the root mean square deviation (RMSD) of their ab initio kinetic trajectories with respect to the optimized structure was simulated. As shown in Figure 14, it can be seen that the RMSD trajectories of the four structures keep rising, proving that the system has not reached the state of energy equilibrium. Between 250 and 1700 fs, the larger the size of pillar[n]arene, the larger the RMSD. Therefore, the larger the molecular size, the more unstable, and after 1700 fs, this rule is broken, and pillar[7]arene becomes a little more stable.

**Figure 15:**
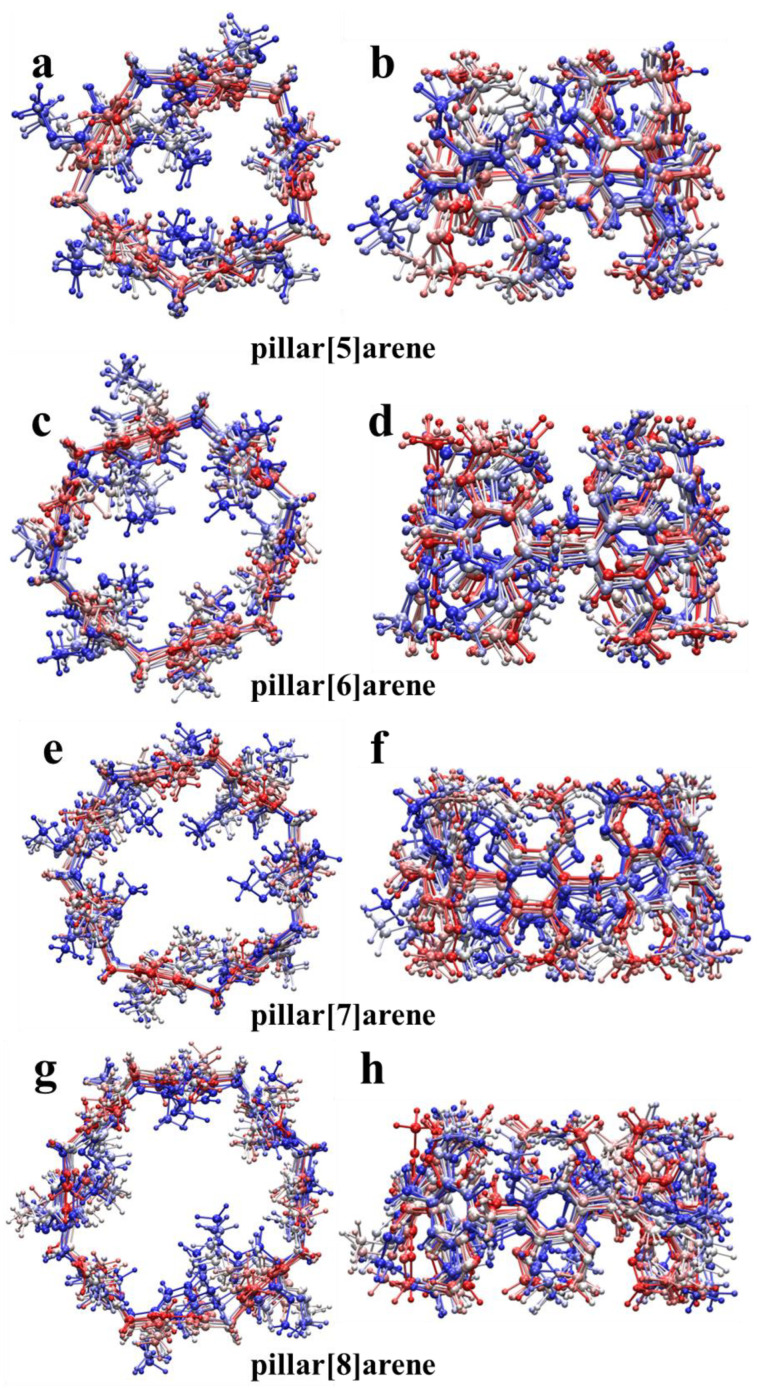
Top (**a**,**c**,**e**,**g**) and main views (**b**,**d**,**f**,**h**) of the structural changes in the 2000 fs trajectory simulated at 298.15 K for pillar[n]arene.

Figure 15 plots the structural changes in the 2000 fs trajectory simulated by pillar[n]arene at a temperature of 298.15 K, with the structure extracted every 200 fs and the red-to-white-to-blue time changes corresponding to the time steps. Pillar[n]arene was found to be structurally stable at a temperature of 298.15 K and no dissociation occurred. It is proved that pillar[n]arene has some stability at 298.15 K.

**Figure 16:**
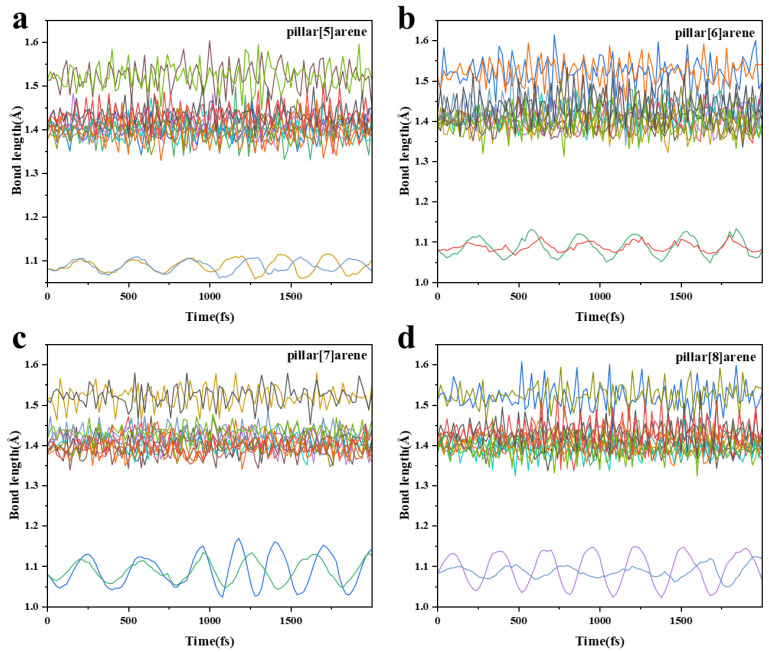
Bond length trajectory of pillar[n]arene at 298.15 K. (**a**): pillar[5]arene; (**b**): pillar[6]arene; (**c**): pillar[7]arene; (**d**): pillar[8]arene. Different colored lines represent different C-C bonds.

We have also simulated the bond length change in the 1,4-dimethoxybenzene in pillar[n]arene at 298.15 K, and the fluctuation in the broken line in the figure represents the degree of bond length oscillation during the simulation. As shown in Figure 16, it can be seen that the C-C bond length oscillation of pillar[7]arene is relatively weak, and the C-O bond oscillation is the strongest. The bond length oscillations of pillar[6]arene and pillar[7]arene are more obvious. Pillar[5]arene has the smallest bond length oscillation, so its structure is relatively stable.

## 3. Materials and Methods

In this work, the gaussview 6.0 software [34] was used to construct the pillar[n]arene model. The Gaussian 16 (A.03) [35] program was used to construct the pillar[n]arene model. The structure was optimized based on the DFT [36] calculation method, using B3LYP functional [37] and 6-311g* basis sets [38] combined with DFT-D3 correction [39]. Based on the optimized structure, the time-dependent density functional theory (TDDFT) calculation method [40], and the CAM-B3LYP functional [41] and 6-311g* basis sets were used for electronic excitation calculation. Based on the Multiwfn [42] program, the excitation results were visualized and analyzed. Origin 2022 software was used to draw the OPA spectrum, TPA spectrum, ECD spectrum, and TMD and Raman spectra of pillar[n]arene [43]. Based on the VMD program [44], the electron–hole density diagram [45], the TEDM, the TMDM, and the ESP [46,47,48,49] of the system were drawn. Vibration modes of pillar[n]arene were plotted by GaussView. The magnetic-induced current density [50] was plotted using the AICD program. The molecular dynamics of pillar[n]arene was simulated by the cp2k program [51], and the visual molecular dynamics (VMD) and Origin programs were used for visual analysis. All calculations in this work were carried out under vacuum conditions.

## 4. Conclusions

To sum up, we constructed pillar[n]arenes of four different sizes. The pore diameters of the four structures are calculated. It is found that as the n number of pillar[n]arene increases, the pore diameters are larger, so the adsorption capacity increases. Through the analysis of its molecular orbitals, it was found that the HOMO-LUMO gap value increased with the increase in molecular size. Therefore, the larger the molecular size, the less likely the molecular electronic transition was to occur. A smaller HOMO-LUMO gap usually means that molecules are more efficient at absorbing electrons. Therefore, this type of molecule may be more efficient in organic optoelectronic devices. Secondly, the physical mechanism of pillar[n]arene’s OPA spectrum, TPA spectrum, and ECD spectrum is studied theoretically. The theoretical studies show that the OPA spectra of pillar[n]arene have two absorption peaks around 180 nm and 260 nm, the absorption peaks are redshifted with the increase in size, and the peak of the absorption peak is significantly increased. The TPA spectra show that with the increase in molecular size, the strong absorption peak was redshifted, and the weak absorption peak blueshifted. On the other hand, the size of the molecule also has a certain influence on the TEDM and TMDM of the system. Theoretical studies have shown that the smaller the size of pillar[n]arene, the stronger the ECD spectrum intensity. So, the size effect can regulate the strength of the ECD spectrum. It is more suitable for drug design and synthetic chemistry. At the same time, Raman spectrum analysis shows that the use of a laser can significantly enhance the Raman intensity of pillar[n]arene. The vibration modes at the highest peak value of the resonance Raman spectra are contraction vibration of the C atom and the O atom and left vibration of the H atom. The vibration mode at the highest peak value of the static Raman spectrum is the stretching vibration of the H atom on the methoxy. By calculating the electrostatic potential, it is found that the larger the molecular size, the less likely the molecular interaction is. The study of magnetic-induced current density found that pillar[n]arene has abundant delocalized π electrons in the benzene ring, which results in a strong response to the external magnetic field. After AIMD analysis, it is proved that pillar[n]arene has some stability at 298.15 K, and the structure of pillar[5]arene is relatively more stable. This study shows that the size effect has a significant regulatory effect on the optical properties of molecules, especially for nanoscale macromolecular systems.

## Figures and Tables

**Table 1:** Main excited state transition index of pillar[n]arene at the main absorption peak near 150–200 nm.

Molecule	Excited States	Oscillator Strength	Excited Energy (eV)	*H* (Å)	*D* (Å)	*t* (Å)	*S_r_*
pillar[5]arene	S_27_	0.7554	7.187	4.828	0.089	−1.958	0.80816
pillar[6]arene	S_31_	0.9971	6.388	5.421	0.003	−3.645	0.87588
pillar[7]arene	S_35_	1.2394	6.414	6.254	0.019	−1.629	0.93473
pillar[8]arene	S_49_	1.5416	6.505	7.115	0.016	−2.691	0.90027

**Table 2:** Transition dipole moments and absorption cross sections of the main TPA-excited states of pillar[n]arene.

Molecule	State	Process	Integral Value (Debye)
pillar[5]arene	S_78_	$\langle \phi_{s0}\left|\mu \right|\phi_{s77}\rangle \times \langle \phi_{s77}\left|\mu \right|\phi_{s78}\rangle$	4.12 × 8.11
pillar[6]arene	S_79_	$\langle \phi_{s0}\left|\mu \right|\phi_{s70}\rangle \times \langle \phi_{s70}\left|\mu \right|\phi_{s79}\rangle$	2.28 × 20.38
pillar[7]arene	S_75_	$\langle \phi_{s0}\left|\mu \right|\phi_{s71}\rangle \times \langle \phi_{s71}\left|\mu \right|\phi_{s75}\rangle$	0.94 × 56.23
pillar[8]arene	S_79_	$\langle \phi_{s0}\left|\mu \right|\phi_{s76}\rangle \times \langle \phi_{s76}\left|\mu \right|\phi_{s79}\rangle$	0.87 × 65.99

**Table 3:** TEDM/TMDM values and tensor product eigenvalues of pillar[n]arene near 190–230 nm.

		X	Y	Z	Eigenvalue
pillar[5]arene	TEDM	−0.0025	−0.0010	2.2078	−17.0793
S_27_	TMDM	0.0020	−0.0018	7.7359	
pillar[6]arene	TEDM	−0.0001	−0.0009	2.5240	−27.0381
S_31_	TMDM	−0.0017	−0.0013	10.7124	
pillar[7]arene	TEDM	0.0009	−0.0008	−2.8084	−38.3265
S_35_	TMDM	−0.0020	0.0038	−13.6471	
pillar[8]arene	TEDM	0.0149	0.0336	−3.0754	−51.0190
S_38_	TMDM	−0.0260	−0.0657	−16.5894	

## Data Availability

Data are contained within the article and Supplementary Materials.

## Supplementary Materials

The following supporting information can be downloaded at: https://www.mdpi.com/article/10.3390/molecules29091912/s1, Figure S1: Schematic diagram of the molecular pore of pillar[n]arene; Figure S2: OPA spectrums of pillar[n]arene; Figure S3: TPA spectrums of pillar[n]arene; Figure S4: pillar[n]aren’s TDM and electron hole pair density (e–h) of absorption peak excited states S3 (a), S2 (b), S2 (c) and S3 (d) near 230 nm–300 nm, respectively; Figure S5: pillar[5]arene two-step transition process in S13, from ground state to intermediate state (c) and from intermediate state to final state (a) TDM and electron hole pair density (d, b); pillar[6]arene two-step transition process in S13, from the ground state to the intermediate state (g) and from the intermediate state to the final state (e) TDM and electron hole pair density (h, f); Figure S6: pillar[7]arene two-step transition process in S15, from ground state to intermediate state (c) and from intermediate state to final state (a) TDM and electron hole pair density (d, b); pillar[8]arene two-step transition process in S17, from the ground state to the intermediate state (g) and from the intermediate state to the final state (e) TDM and electron hole pair density (h, f); Figure S7: ECD spectra of pillar[n]arene; Figure S8: The TEDM and TMDM (a–c) of the major ECD excited states of pillar[5]arene near 150–190 nm. The TEDM and TMDM (d–f) of the major ECD excited states of pillar[6]arene near 150–190 nm; Figure S9: The TEDM and TMDM (a–c) of the major ECD excited states of pillar[7]arene near 150–190 nm. The TEDM and TMDM (d–f) of the major ECD excited states of pillar[8]arene near 150–190 nm; Figure S10: The vibration pattern of pillar[5]arene at the peak of Raman absorption; Figure S11: The vibration pattern of pillar[6]arene at the peak of Raman absorption; Figure S12: The vibration pattern of pillar[7]arene at the peak of Raman absorption; Figure S13: The vibration pattern of pillar[8]arene at the peak of Raman absorption; Table S1: pillar[n]arene main excited state transition index of absorption peak near 230 nm–300 nm; Table S2: pillar[n]arene’s transition dipole moment and absorption cross section of two-photon absorption excited state at 400–450 nm; Table S3: TEDM/TMDM values and tensor product eigenvalues of pillar[n]arene near 150–190 nm.
