# Supplementary material for: Quantum Size-Driven Spectral Variations in Pillar[n]arene Systems: A Density Functional Theory and Wave Function Assessment
Source: Molecules. 2024 Apr 23;29(9):1912. doi: 10.3390/molecules29091912 (PMC11085802; doi:10.3390/molecules29091912)
Supplement: Supplementary file 1 [file molecules-29-01912-s001.zip › molecules-2969269-supplementary.pdf]

**Supporting information**

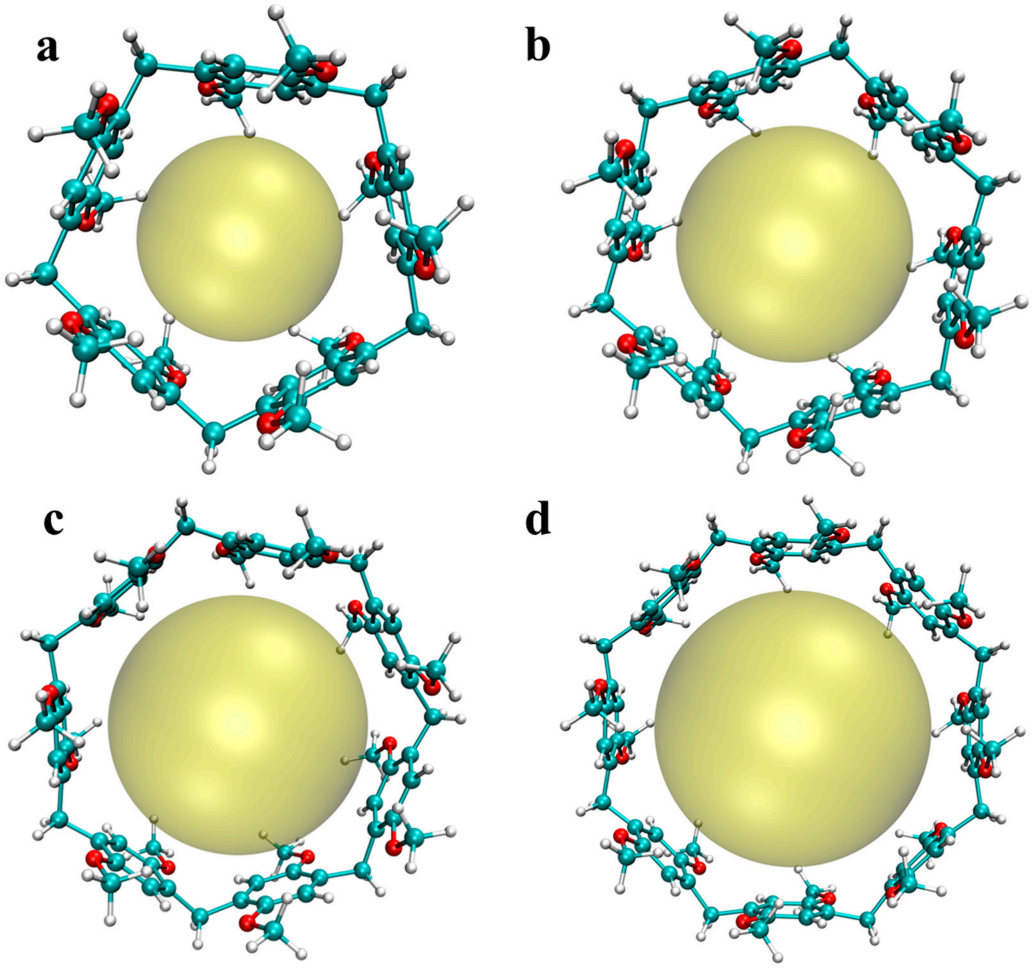


Figure S1: Schematic diagram of the molecular pore of pillar[n]arene.

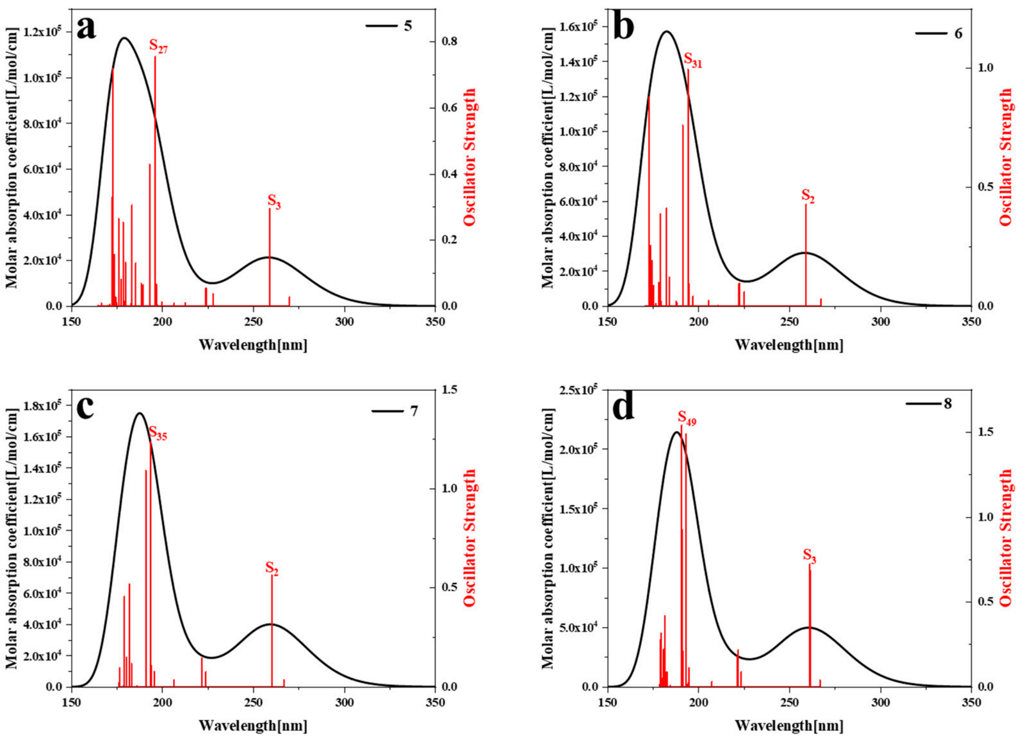


Figure S2: OPA spectrums of pillar[n]arene.

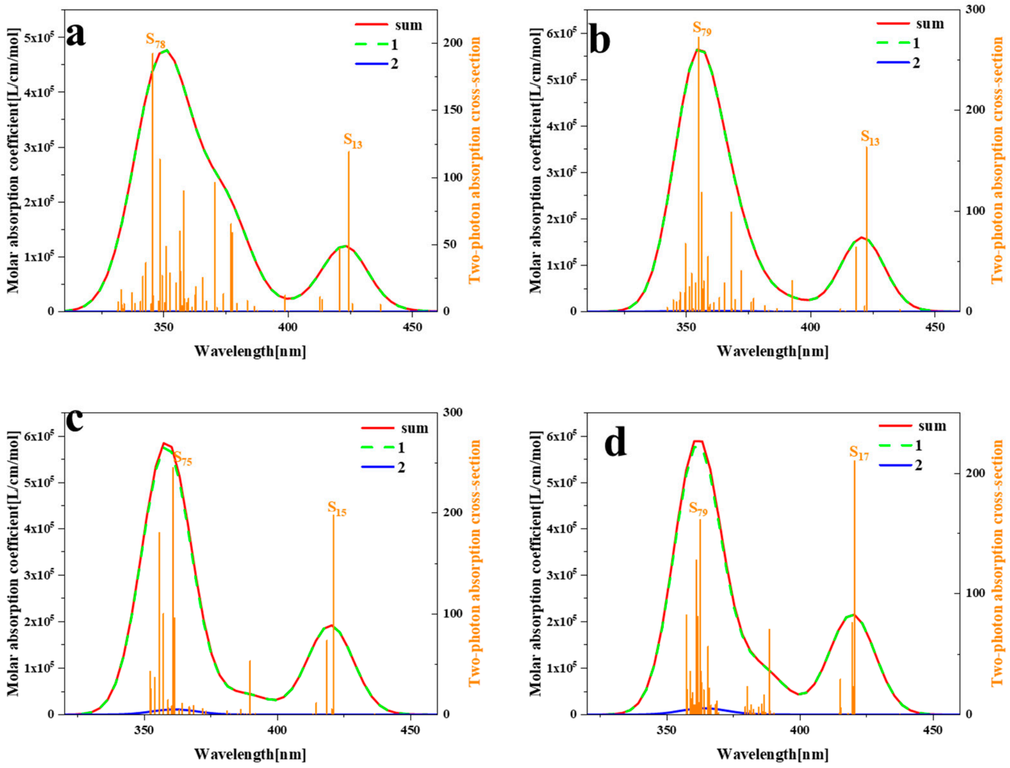


Figure S3: TPA spectrums of pillar[n]arene.

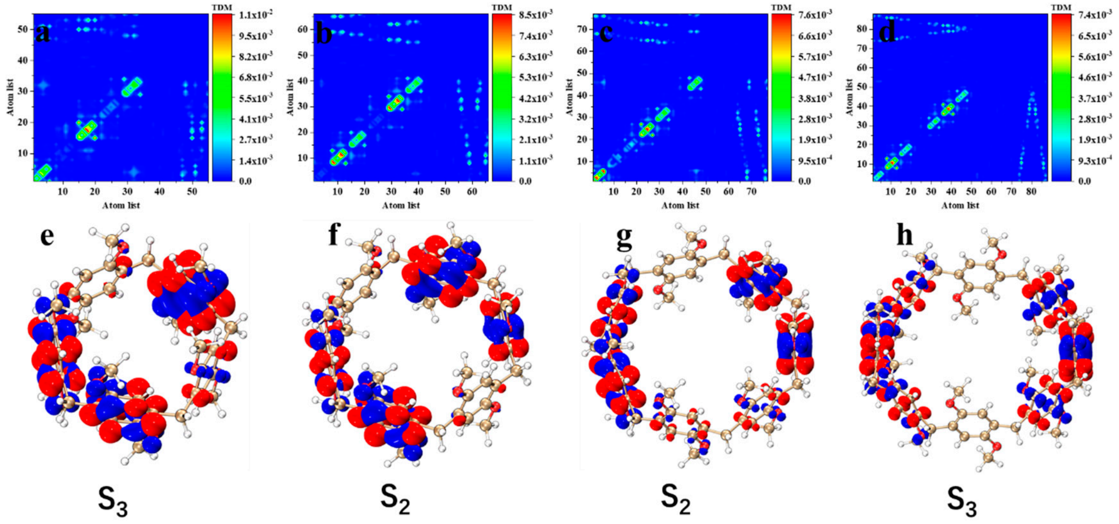


Figure S4. pillar[n]aren's TDM and electron hole pair density (e-h) of absorption peak excited states S3(a), S2(b), S2(c) and S3(d) near 230nm-300nm, respectively.

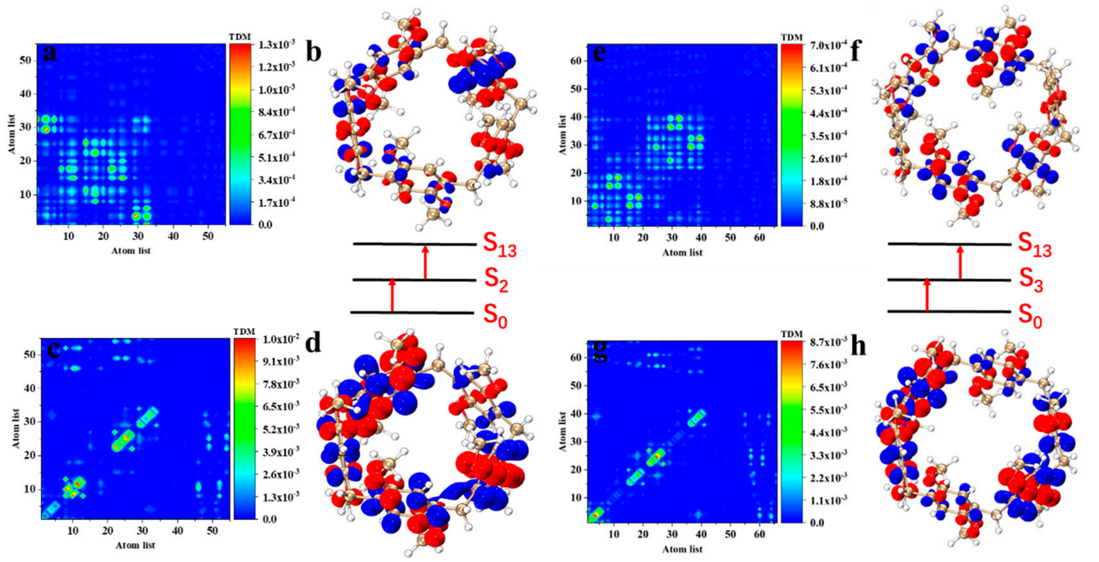


FigureS5. pillar[5]arene two-step transition process in $S_{13}$, from ground state to intermediate state (c) and from intermediate state to final state (a) TDM and electron hole pair density (d, b); pillar[6]arene two-step transition process in $S_{13}$, from the ground state to the intermediate state (g) and from the intermediate state to the final state (e) TDM and electron hole pair density (h, f).

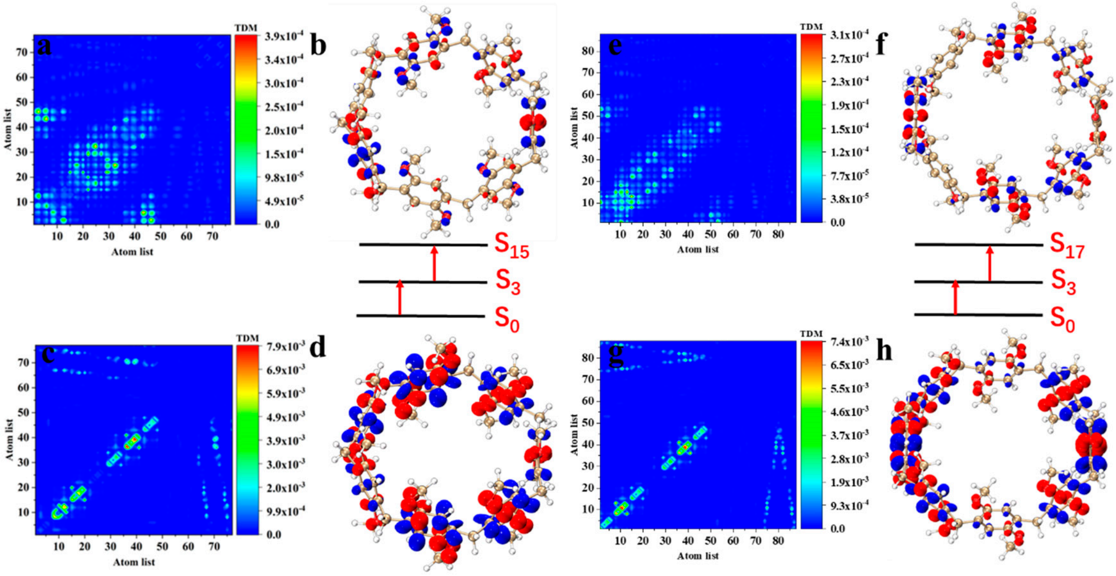


FigureS6. pillar[7]arene two-step transition process in $S_{15}$, from ground state to intermediate state (c) and from intermediate state to final state (a) TDM and electron hole pair density (d, b); pillar[8]arene two-step transition process in $S_{17}$, from the ground state to the intermediate state (g) and from the intermediate state to the final state (e) TDM and electron hole pair density (h, f).

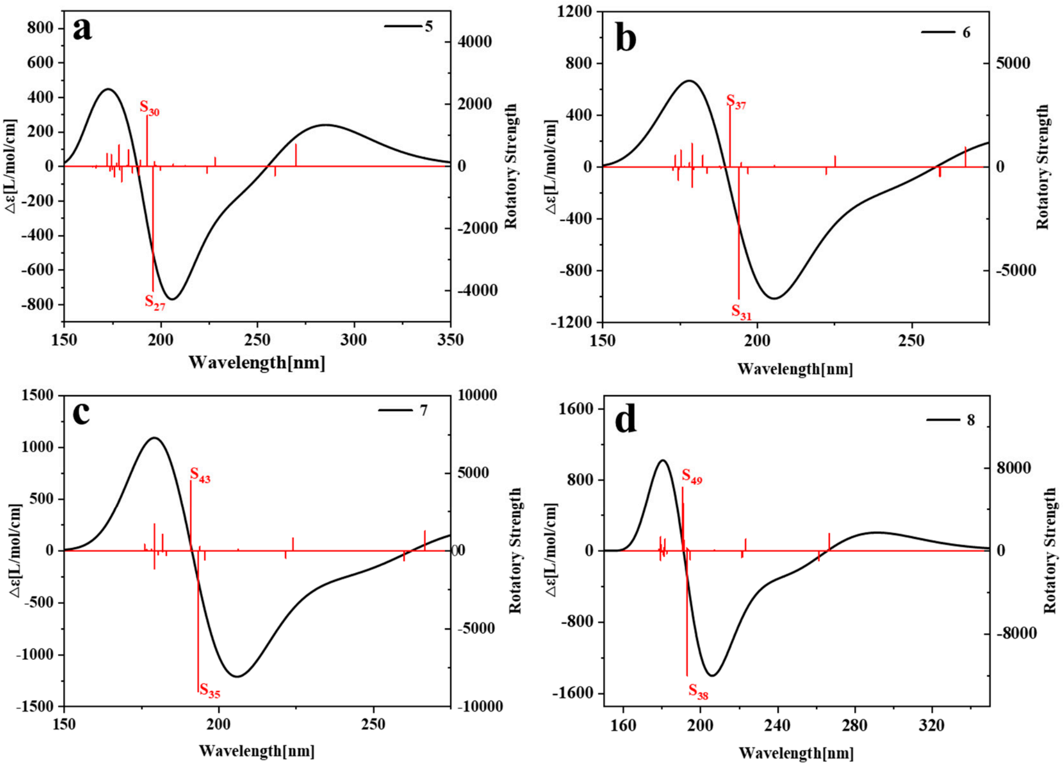


Figure S7. ECD spectra of pillar[n]arene.

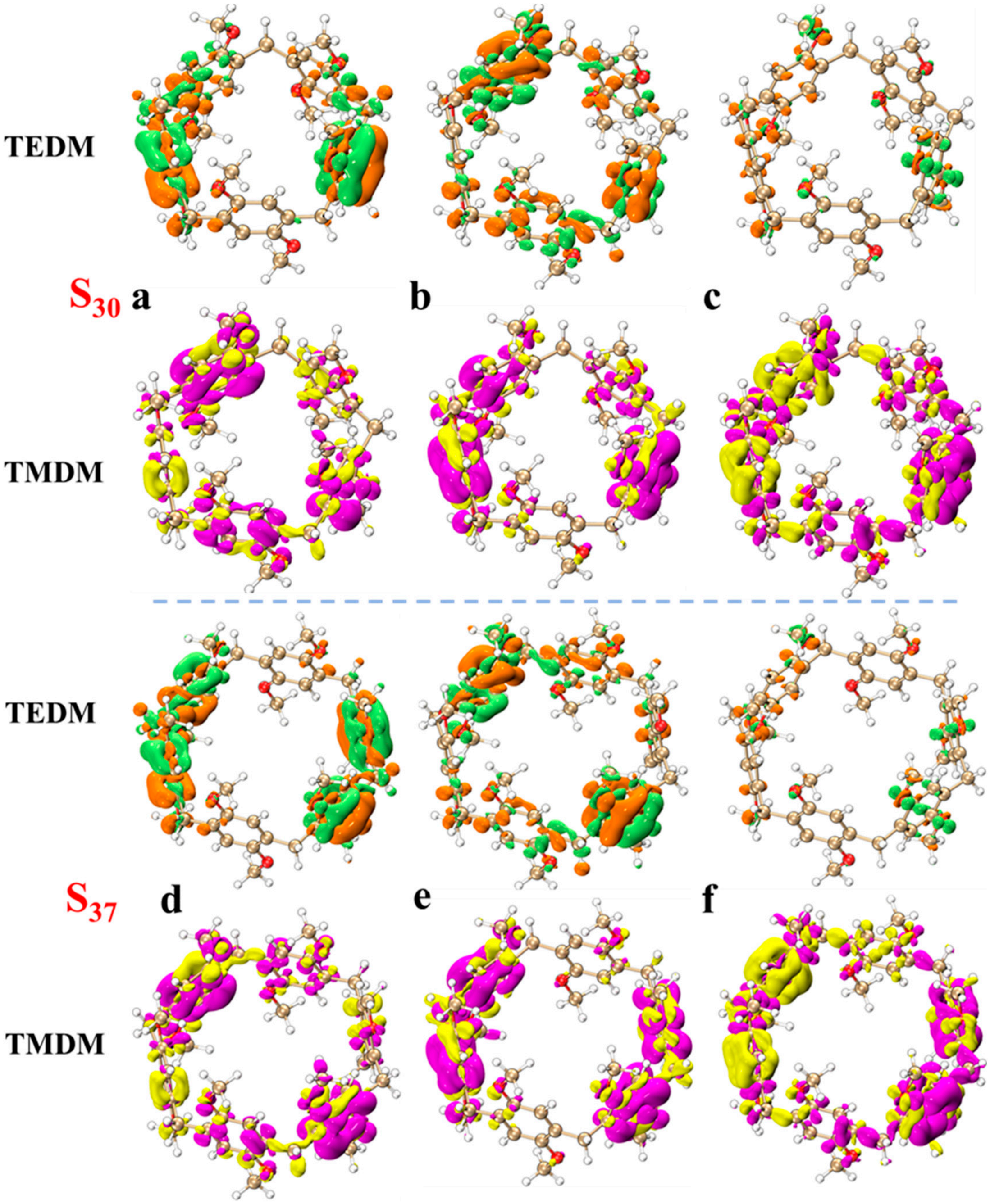


Figure S8. The TEDM and TMDM (a-c) of the major ECD excited states of pillar[5]arene near 150-190nm. The TEDM and TMDM (d-f) of the major ECD excited states of pillar[6]arene near 150-190nm.

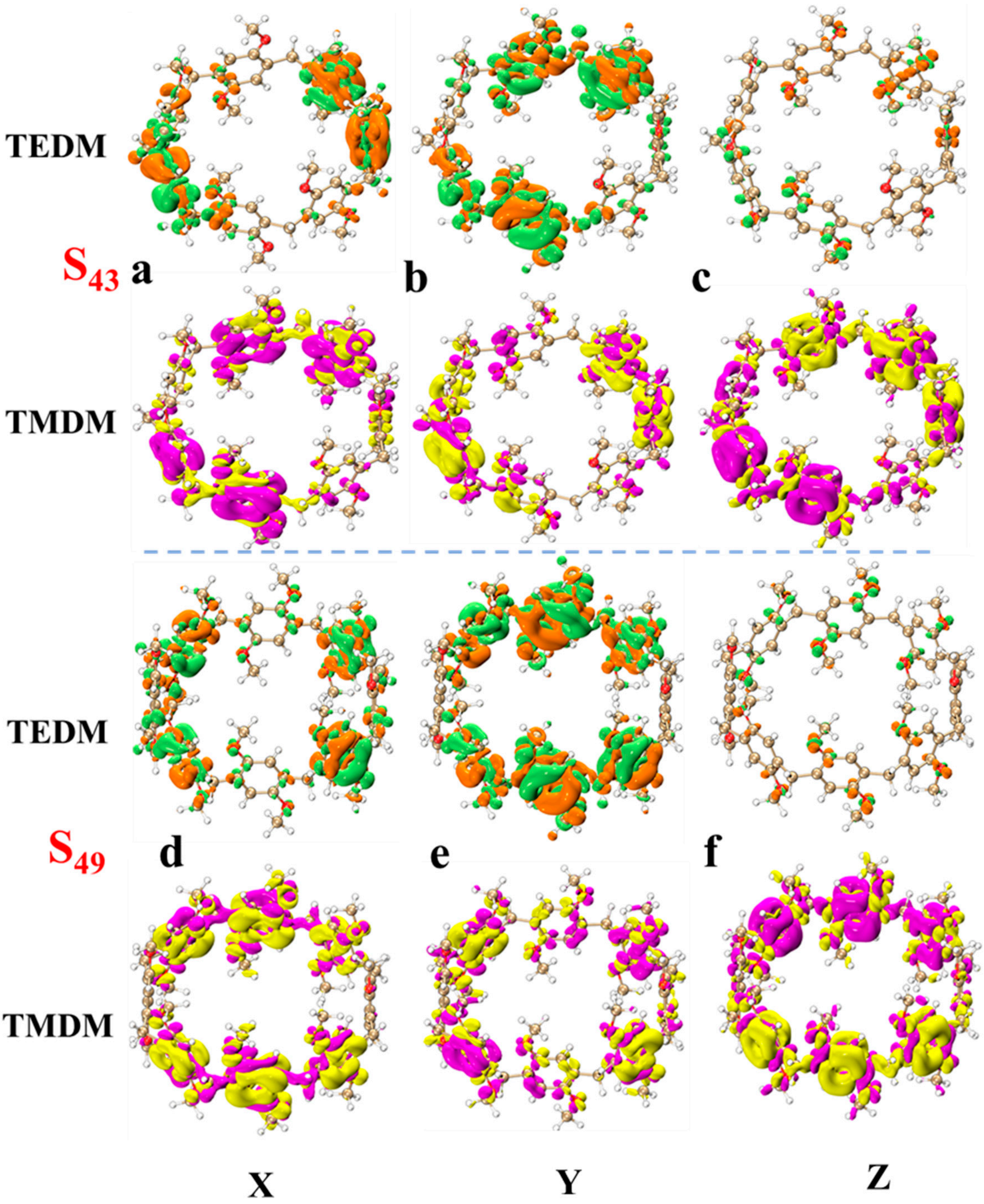


Figure S9. The TEDM and TMDM (a-c) of the major ECD excited states of pillar[7]arene near 150-190nm. The TEDM and TMDM (d-f) of the major ECD excited states of pillar[8]arene near 150-190nm.

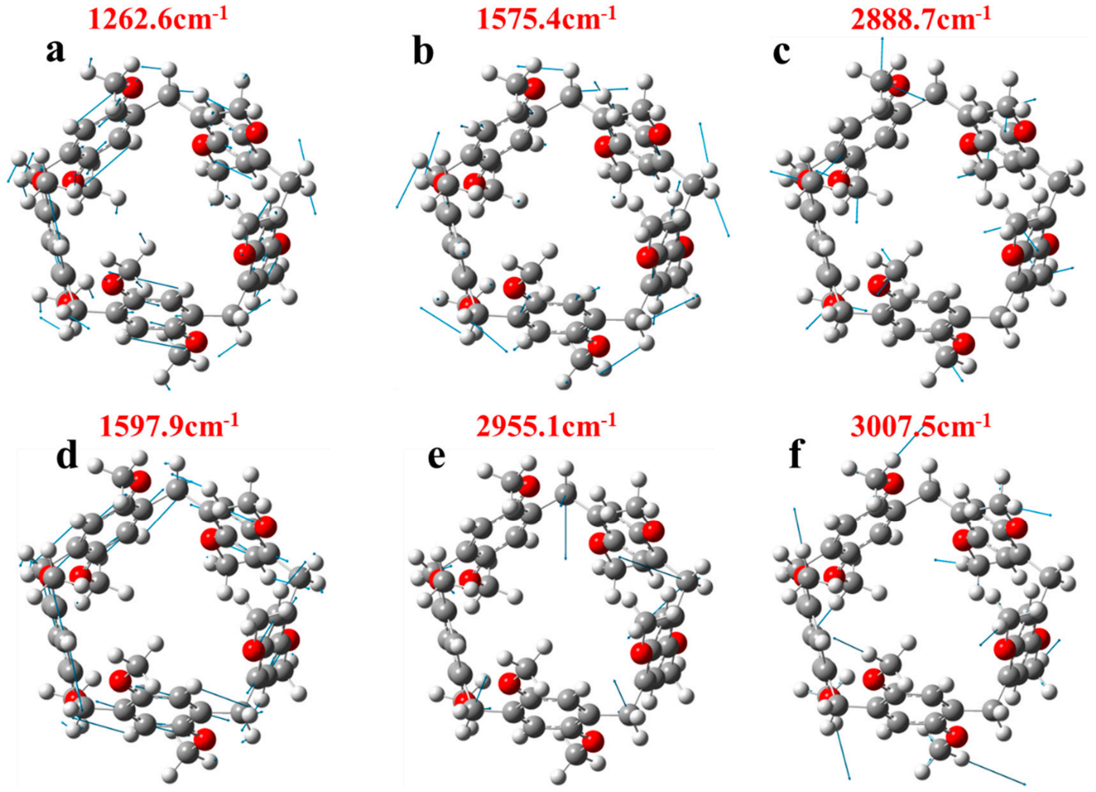


Figure S10. The vibration pattern of pillar[5]arene at the peak of Raman absorption.

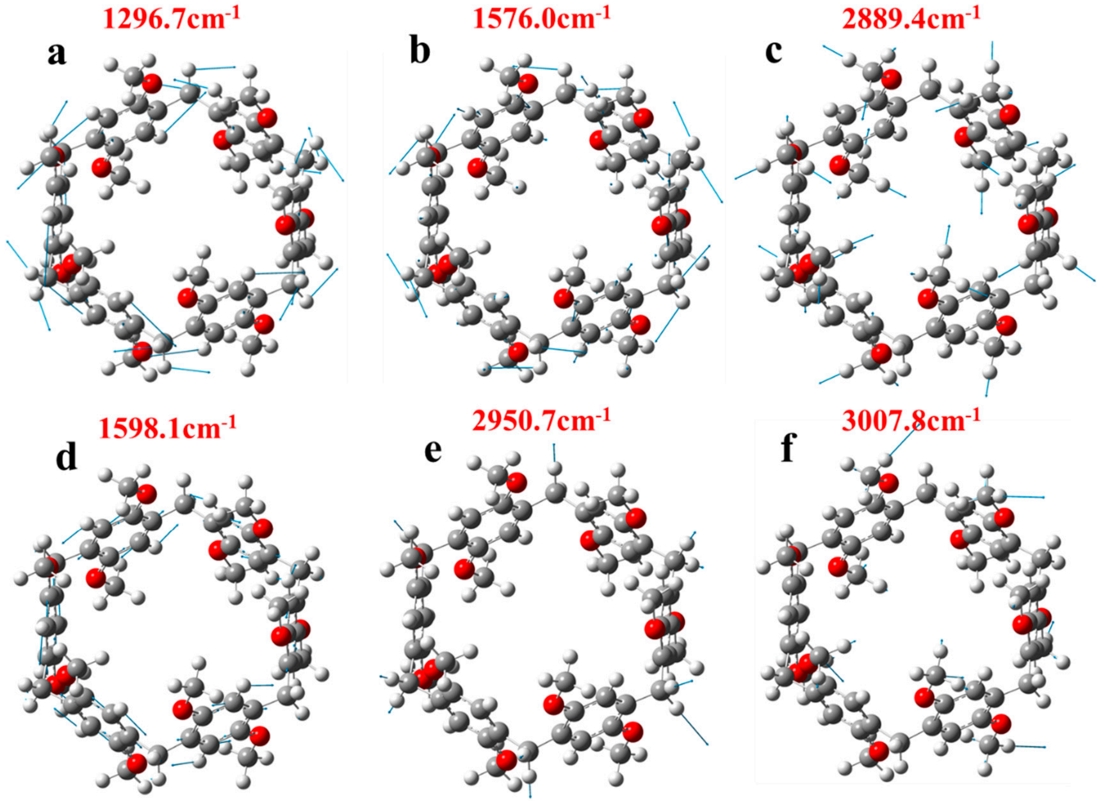


Figure S11. The vibration pattern of pillar[6]arene at the peak of Raman absorption.

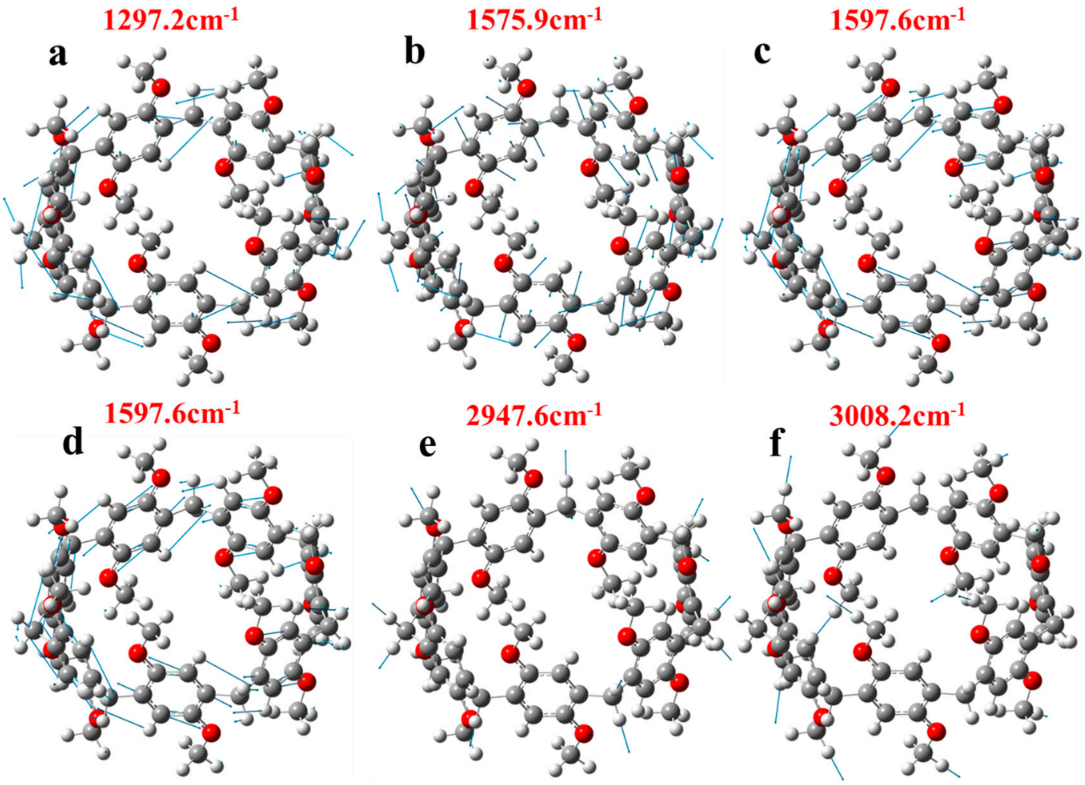


Figure S12. The vibration pattern of pillar[7]arene at the peak of Raman absorption.

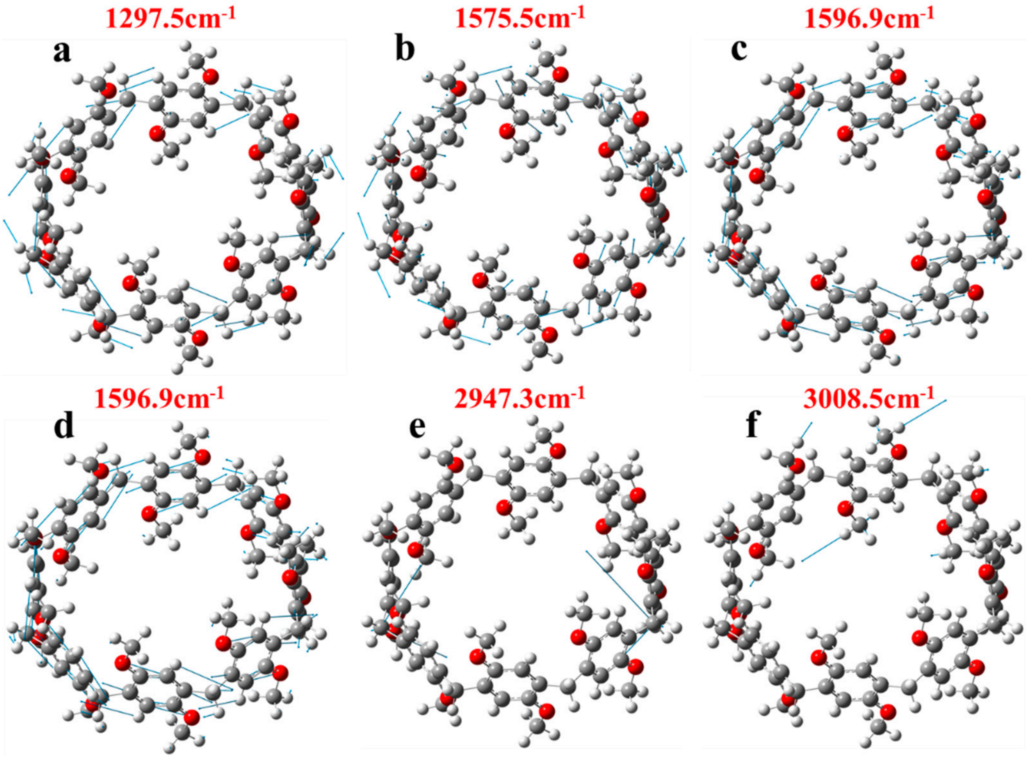


Figure S13. The vibration pattern of pillar[8]arene at the peak of Raman absorption.

Table S1. pillar[n]arene main excited state transition index of absorption peak near 230nm-300nm.

| Molecule | Excited states | Oscillator Strength | Excited energy(eV) | H(Å) | D(Å) | t (Å) | Sr |
|---|---|---|---|---|---|---|---|
| pillar[5]arene | $S_3$ | 0.2959 | 4.788 | 4.614 | 0.005 | -1.548 | 0.81032 |
| pillar[6]arene | $S_2$ | 0.4271 | 4.786 | 5.430 | 0.008 | -3.524 | 0.80260 |
| pillar[7]arene | $S_2$ | 0.5653 | 4.769 | 6.263 | 0.002 | -2.226 | 0.79582 |
| pillar[8]arene | $S_3$ | 0.6880 | 4.749 | 7.105 | 0.003 | -5.042 | 0.79208 |

Table S2. pillar[n]arene's transition dipole moment and absorption cross section of two-photon absorption excited state at 400-450nm.

| Molecule | State | Process | Integral value(Debye) |
|---|---|---|---|
| pillar[5]arene | S13 | $\langle \phi_{s0} \lvert \mu \rvert \phi_{s2} \rangle \times \langle \phi_{s2} \lvert \mu \rvert \phi_{s13} \rangle$ | 2.57×2.75 |
| pillar[6]arene | S13 | $\langle \phi_{s0} \lvert \mu \rvert \phi_{s3} \rangle \times \langle \phi_{s3} \lvert \mu \rvert \phi_{s13} \rangle$ | 3.71×2.43 |
| pillar[7]arene | S15 | $\langle \phi_{s0} \lvert \mu \rvert \phi_{s3} \rangle \times \langle \phi_{s3} \lvert \mu \rvert \phi_{s15} \rangle$ | 4.93×2.11 |
| pillar[8]arene | S17 | $\langle \phi_{s0} \lvert \mu \rvert \phi_{s3} \rangle \times \langle \phi_{s3} \lvert \mu \rvert \phi_{s17} \rangle$ | 6.36×1.91 |

Table S3 TEDM/TMDM values and tensor product eigenvalues of pillar[n]arene near 150-190nm.

| | | X | Y | Z | Eigenvalue |
|---|---|---|---|---|---|
| pillar[5]arene | TEDM | -0.7275 | -1.4806 | -0.0012 | 6.9575 |
| S30 | TMDM | 1.8640 | 3.7832 | -0.0022 | |
| pillar[6]arene | TEDM | -1.0943 | -1.8896 | -0.0005 | 12.7010 |
| S37 | TMDM | 2.9121 | 5.0351 | -0.0038 | |
| pillar[7]arene | TEDM | -1.9339 | 1.7687 | -0.0001 | 19.3562 |
| S43 | TMDM | 5.4511 | -4.9835 | -0.0008 | |
| pillar[8]arene | TEDM | 3.1032 | 0.2103 | 0.0024 | 26.3702 |
| S49 | TMDM | -8.4583 | -0.5823 | 0.0178 | |
